# Internal and external microbiota of home-caught *Anopheles coluzzii* (Diptera: Culicidae) from Côte d’Ivoire, Africa: Mosquitoes are filthy

**DOI:** 10.1371/journal.pone.0278912

**Published:** 2022-12-15

**Authors:** Kaiying Chen, Loganathan Ponnusamy, Chouaïbou S. Mouhamadou, Behi Kouadio Fodjo, Gba Christabelle Sadia, France Paraudie Kouadio Affoue, Jean M. Deguenon, R. Michael Roe

**Affiliations:** 1 Department of Entomology and Plant Pathology, College of Agriculture and Life Sciences, North Carolina State University, Raleigh, NC, United States of America; 2 State Key Laboratory for Biology of Plant Diseases and Insect Pests, Institute of Plant Protection, Chinese Academy of Agricultural Sciences, Beijing, China; 3 Comparative Medicine Institute, North Carolina State University, Raleigh, NC, United States of America; 4 Centre Suisse de Recherches Scientifiques, Abidjan, Cote d’Ivoire, Africa; Uppsala University: Uppsala Universitet, SWEDEN

## Abstract

Over the past 10 years, studies using high-throughput 16S rRNA gene sequencing have shown that mosquitoes harbor diverse bacterial communities in their digestive system. However, no previous research has examined the total bacteria community inside versus outside of mosquitoes and whether bacteria found on the outside could represent a potential health threat through mechanical transfer. We examined the bacterial community of the external surface and internal body of female *Anopheles coluzzii* adults collected from homes in Côte d’Ivoire, Africa, by Illumina sequencing of the V3 to V4 region of 16S rRNA gene. *Anopheles coluzzii* is in the *Anopheles gambiae* sensu lato (s.l.) species complex and important in the transmission of malaria. The total 16S rRNA reads were assigned to 34 phyla, 73 orders, 325 families, and 700 genera. At the genus level, the most abundant genera inside and outside combined were *Bacillus*, *Staphylococcus*, *Enterobacter*, *Corynebacterium*, *Kocuria*, *Providencia*, and *Sphingomonas*. Mosquitoes had a greater diversity of bacterial taxa internally compared to the outside. The internal bacterial communities were similar between homes, while the external body samples were significantly different between homes. The bacteria on the external body were associated with plants, human and animal skin, and human and animal infections. Internally, *Rickettsia bellii* and *Rickettsia typhi* were found, potentially of importance, since this genus is associated with human diseases. Based on these findings, further research is warranted to assess the potential mechanical transmission of bacteria by mosquitoes moving into homes and the importance of the internal mosquito microbiota in human health.

## Introduction

Insects and other arthropods are found in nearly all habitats, are extraordinarily adaptable, and produce large numbers of progeny in a relatively short time [1]. They are able to transmit bacteria, fungi, viruses, and protozoa by mechanical transmission (physical contact) or by biological transmission (from inside their body to another host) [2, 3]. In biological transmission, some of the pathogens relocate within the body of the vector, moving from the digestive system to the salivary glands; for example, *Rickettsia rickettsii* that causes Rocky Mountain spotted fever, is dormant in the tick’s body and activated after the initiation of tick feeding [4]. In mechanical transmission, house flies that live in filth and garbage carry and transmit on their legs and mouthparts, the pathogens for cholera, typhoid fever, and dysentery [5–7]. Acute respiratory syndrome coronaviruses (SARS-CoV) were reported to be mechanically transmitted by insects [8, 9].

Mosquitoes play a significant role in the transmission of diseases like Malaria, Zika fever, Dengue, Chikungunya, and Yellow fever. Of the medically important mosquito species, *Anopheles gambiae* sensu lato (s.l.) [10], largely anthropophilic, is an important malaria vector in Africa; the disease is caused by a parasitic protozoan of the genus *Plasmodium*. Globally in 2020, an estimated 241 million malaria cases were reported [11]. The WHO African Region accounted for 95% of these cases and 96% of the deaths with 85% being children under 5 years old [11].

Vector control is a highly efficacious prevention method for malaria. The main vector control measures are indoor residual sprays (IRSs) and insecticide-treated nets (ITNs). IRSs and ITNs can decrease the vectorial capacity by reducing the adult’s longevity, density, and biting rate per day. However, the development of insecticide resistance has decreased their efficacy [12]. Recent survey data showed both *Anopheles coluzzii* (one species of the *An*. *gambiae* complex) and *An*. *gambiae* sensu stricto (s.s.) had high resistance levels to pyrethroid, organochlorine, and carbamate insecticides [13]. New strategies for managing resistance and malaria vector control are needed.

In the last 10 years, microbiota studies have been conducted on *Anopheles* mosquitoes from African countries, including Kenya [14–16], Ghana [17], Mali [18], Ethiopia [19], Burkina Faso [16, 20, 21], Cameron [22, 23] and the Republic of Guinea [16]. The bacterial microbiota of *Anopheles* mosquitoes has also been characterized in other malaria-endemic Asian countries, i.e., Vietnam [24], Thailand [25], and India [26]. Several recent microbiota studies on the mosquito midgut have revealed the presence of a diverse microbiome, which can significantly affect the development, digestion, immunity, metabolism, and other physiological functions of the mosquito [27, 28]. Additionally, the microbiome affects the ability of insects to transmit pathogens. For example, the mosquito’s midgut microbiota was involved in the suppression of *Plasmodium falciparum* by stimulating the basal immune response of *An*. *gambiae* s. s. mosquitoes [29, 30]. Although microbiomes have been studied in many different mosquito species, our understanding of bacterial composition and structure in *An*. *coluzzii* is limited. Further, mosquitoes are exposed to a range of microbes according to their ecological niches like larval habitats [31], outdoor resting sites [32], and plant nectar sources [33]. This could play an essential role in malaria transmission in different environments. Characterizing the bacterial community of *An*. *gambiae* s. l. in homes in Côte d’Ivoire will improve our understanding of their microbiome in the human domestic environment. Additionally, like with filth flies, *Anopheles* mosquitoes moving from outside into homes, and interacting with surfaces in the home and people directly has the potential of transmitting bacteria mechanically that could be a health concern different from the well recognized biological transmission by mosquitoes. To our knowledge, this is the first study conducted to investigate the external body bacteria of *An*. *gambiae* s.l. and whether *Anopheles* mosquitoes are potential mechanical vectors.

The objective of the study is to investigate the external and internal body bacterial communities of home-caught adult female *An*. *coluzzii* using culture-independent methods and providing a basis for future studies characterizing the biological importance of bacteria in this species. Based on previous studies by Deguenon et al. [6], we hypothesize that the bacterial communities from the external and internal body of the mosquito possibly vary, and the bacterial communities from the external surface of the mosquito body between homes would be diverse. Further, we hypothesized that the mosquitoes collected from homes in Africa might harbor pathogenic bacteria not previously appreciated.

## Material and methods

### Ethics statement

All persons in visited households were informed about the purpose and nature of the study, what participation in the study requires, possible risks and benefits, and verbal consent was obtained and witnessed by signature on a master household list. It was also stressed that any person may refuse dwelling mosquito search/collection at any time without negative consequences. None of the information registered is sensitive. The master household list included a unique identification # per house. Household names and GPS locations were not recorded. All results of the study were reported blindly.

### Mosquito sampling and sample preparation

In June 2019, adult mosquitoes were collected in the municipality of Tiassalé, Africa, in the south of Côte d’Ivoire about 110 km North of Abidjan, one of the country’s largest cities [34]. Rice production occurs in the Tiassalé’s lowlands, allowing mosquitos to increase all year, and malaria is the primary cause of sickness [34]. Sampling was conducted house-by-house in a five-block area of Tiassalé (5°53’54" N, 4°49’42" W; S1 Fig). A total of 80 houses were visited, and all the adult mosquitoes in the living rooms were collected. A total of 79 female mosquitoes were collected from 9 houses (see S1 File; most of the homes did not have mosquitoes). Female mosquitoes were collected using aspirators. Mosquitoes collected from each home were placed in a new 125 mL plastic cup. Less than 2 hours after collection, the samples were transported from the field back to the lab. They were chilled in freezer for about 45 seconds, gently picked up with forceps on one leg, and placed on a microscope slide to identify species based on their morphology [10]. After identification, mosquitos were placed individually into 1.5 ml microcentrifuge tubes containing 200 μL of RNAlater and shipped to the North Carolina State University for DNA analysis. Each mosquito was inspected for intactness (all body parts attached), and then intact mosquitoes were rinsed four times with 200 μL of sterile 0.01 M phosphate-buffered saline (PBS, pH 7.4 at 25°C). All wash solutions and RNAlater were pooled for each mosquito and stored at -80°C for later surface microbiome analysis (external body samples). The mosquitoes were then stored in 100% ethanol at 4°C until further use.

### DNA extraction and mosquito molecular identification

Before DNA extraction, each mosquito was sterilized with 1% bleach for 30 s, followed by five separate washes with 200 μL PBS (pH 7.4 at 25°C). The last wash of each mosquito was kept for further verification of the success of the surface sterilization. After surface sterilization, mosquitoes were transferred individually to sterile 2 mL screw-capped microcentrifuge tubes containing glass beads with 200 μL of TNE buffer (100 mM Tris, 0.2 M NaCl, 10 mM EDTA, pH 7.4) added, and samples were homogenized using a FastPrep FP120 cell homogenizer (Thermo Electron Corporation, Waltham, MA, USA). The external body samples were lyophilized to dryness and resuspended in 200 μL of TNE buffer. The DNA of internal body and external body samples were extracted separately from each mosquito using the QIAGEN DNeasy Blood & Tissue Kit based on the manufacturers’ instructions (QIAGEN, Valencia, CA, USA). DNA quality and quantity were assessed using a Nanodrop (Thermo Fisher Scientific, Waltham, Massachusetts), DNA concentration normalized to 50 ng/μL, and then samples stored at -40°C until PCR amplification. *An*. *gambiae* complex mosquitoes species were molecularly identified (M or S form) using amplicons size of Short Interpersed Element (SINE) regions by PCR, using previously published primers [35, 36]. SINE-PCR was amplified using 2 μL of DNA template extracted from individual mosquito, 12.5 μL of AmpliTaq Gold™ 360 Master Mix, 1μL (10 pmol) of primer F6.1A, 1μL (10 pmol) of primer R6.1B, and molecular grade nuclease-free water added to achieve a final volume of 25 μL. The temperature of the PCR reaction was set as follows: 95°C for 10 min, 35 cycles of 95°C for 30 s, 54°C for 30 s and 72°C for 30 s, then 72°C for 10 min. Amplified PCR product was visualized in a 1.5% electrophoresis gel stained by ethidium bromide under UV light. To further verify sequence of the PCR products, 10% of the PCR products (eight samples) were chosen randomly and sequenced by Eton Bioscience (Research Triangle Park, NC, USA).

### Illumina library preparation and sequencing

A total of 157 16S V3–V4 amplicon libraries (79 internal body and 78 external body) were prepared according to the Illumina metagenomic sequencing library construction workflow. Briefly, universal 16S primers (341F/806R) were used to amplify the hypervariable V3-V4 region of the bacterial 16S rRNA genes [37]. DNA extraction from the pooled last washes yielded no bands and were not submitted for sequencing. The target amplicon and index PCR product was purified using AMPure XP beads (AXYGEN, Big Flats, NY, USA). Additionally, four mock community gDNA standard (D6305, ZymoBIOMICS™, Irvine, CA) amplicon libraries were included as positive controls to determine PCR amplification bias or sequencing error [38]. DNA library concentration was measured with Quant-iT PicoGreen (Molecular Probes, Inc. Eugene, OR, USA). Final libraries were pooled in equimolar amounts. Illumina sequencing (300-bp paired-ends) was performed at the Microbiome Core Facility, School of Medicine, University of North Carolina, Chapel Hill, NC, USA.

### Bioinformatics data processing and statistical analyses

Illumina FASTQ files were demultiplexed and quality-filtered (q20) using Quantitative Insights into Microbial Ecology 2 (QIIME2) [39]. Reads were then denoised, and the paired-end reads merged with the chimera removed using the DADA2 plugin [40]. Primer sequences were trimmed (--p-trim-left-f 17,--p-trim-left-r 21), and the forward and reverse sequences were truncated at 290 and 280 nucleotide, respectively, to remove low-quality sequences (--p-trunc-len-f 290, --p-trunc-len-r 280). DADA2 replaces the traditional OTU-picking process, and it models the sequence error and constructs the exact biological sequences in the samples called Amplicon Sequence Variants (ASVs). Sequences were aligned with the *Align-to-tree-mafft-fasttree* pipeline, and a phylogeny tree was constructed with *q2-phylogeny*. Taxonomy classification was performed on representative sequences that were generated from DADA2 using a Naive Bayes classifier from Greengenes [41] 13_8 with 99% sequence similarity to the OTU data set, then trained following QIIME 2 tutorial docs “Training feature classifiers with q2-feature-classifier” (https://docs.qiime2.org/2022.8/tutorials/feature-classifier/). The sequences of the taxa with relative abundance higher than 1% have also been extracted for BLASTn searches against the National Center for Biotechnology Information (NCBI) database (http://blast.ncbi.nlm.nih.gov/Blast.cgi).

Additionally, the q2–diversity plugin pipeline was used to conduct the alpha and beta diversity analysis (--p-sampling-depth 8244). Alpha diversity (which estimates diversity within samples) was measured by Shannon’s diversity index [42], Observed Features and Faith’s Phylogenetic Diversity [43]. To examine the effects of internal body versus external body and sampling locations (different homes), the non-parametric Kruskal-Wallis test (α = 0.05) was used on alpha diversity. Beta diversity was measured by the weighted UniFrac [44] and Bray Curtis distance [45], and two of the households with only one mosquito each were excluded. Further, two households with three specimens were excluded from Beta diversity analysis to test whether sample size plays a role in statistical power using Unifrac and Bray Curtis. A PERMANOVA test was run to test for statistical differences between the internal body and external body samples, among sampling homes of internal body samples; and the statistical difference among sampled homes for external body samples. EMPeror [46] was used to visualize the principal coordinate analysis (PCoA) plots from the beta diversity analyses. To see if there was a difference in the abundance of common bacterial taxa between the internal body and external body, t-tests (assuming unequal variances, α = 0.05) were performed using the JMP® Pro 16 software (SAS Institute Inc., Cary, NC, USA). The ANCOM [47, 48] analysis was performed using QIIME2 to test the differentially abundant taxa across different homes.

### Phylogenetic analyses of *Rickettsia* sequences

Approximate phylogenetic relationships were examined for nine *Rickettsia* sequence variants from this study with other *Rickettsia* sequences obtained from the NCBI database by BLASTn analysis (accessed on January 18, 2021). Multiple alignments were performed using the ClustalW program [49]. Phylogenetic trees were constructed using the maximum likelihood (ML) and neighbor-joining (NJ) analyses with the Kimura two-parameter model [50] in MEGA 11 software [51]. Bootstrapping at 1000 re-sampling iterations were calculated for ML and NJ trees.

## Results

### Mosquitoes identification

The SINE gene (~ 479 bp band) was successfully amplified for all 79 female *An*. *gambiae* complex mosquitoes. Based on the visualization of PCR products resolved on agarose gels, all PCR products appeared as a single band, and there was no measurable length variation among samples. BLAST results for the sequences showed 99.50 to 99.75% similarity to *An*. *gambiae* M from Cameroon SINE S200X6.1 (GenBank Accession number EU881873). Thus, all the mosquitoes collected in this study were *An*. *coluzzii*, formerly known as *An*. *gambiae* M form [52].

### Evaluation of the mock microbial community

The mock microbial community consisted of 8 bacterial species from 8 distinct genera (S1 Table). All the species present in the mock community were identified by ASV assembly and taxonomic classification. In addition, the ASV assembly and taxonomic classification identified an additional bacterial genus, *Methylobacterium*, which was not present in the mock community. Due to their low relative abundance and no other plausible explanations for their detection, we concluded these were likely either contaminant from sample processing or barcode cross talk from sequencing. Thus, *Methylobacterium* was excluded from further analysis.

### Data summary of bacterial sequences from *Anopheles coluzzii*

Illumina sequencing of 16S rRNA produced abundant reads for the bacterial communities across different homes where mosquitoes were collected. A total of 34,221,107 sequence reads were obtained across the 157 (79 internal and 78 external) samples. After quality filtering with the DADA2 algorithm, 17,267,125 reads were obtained with an average of 109,981.69 reads *per* sample and were assigned to 34 phyla, 73 orders, 325 families, and 700 genera. After rarefaction analysis, four samples (three internal and one external) with lower sequencing depth (less than 8244 sequencing reads) were discarded from the diversity analysis.

### Alpha diversity

**Fig 1:**
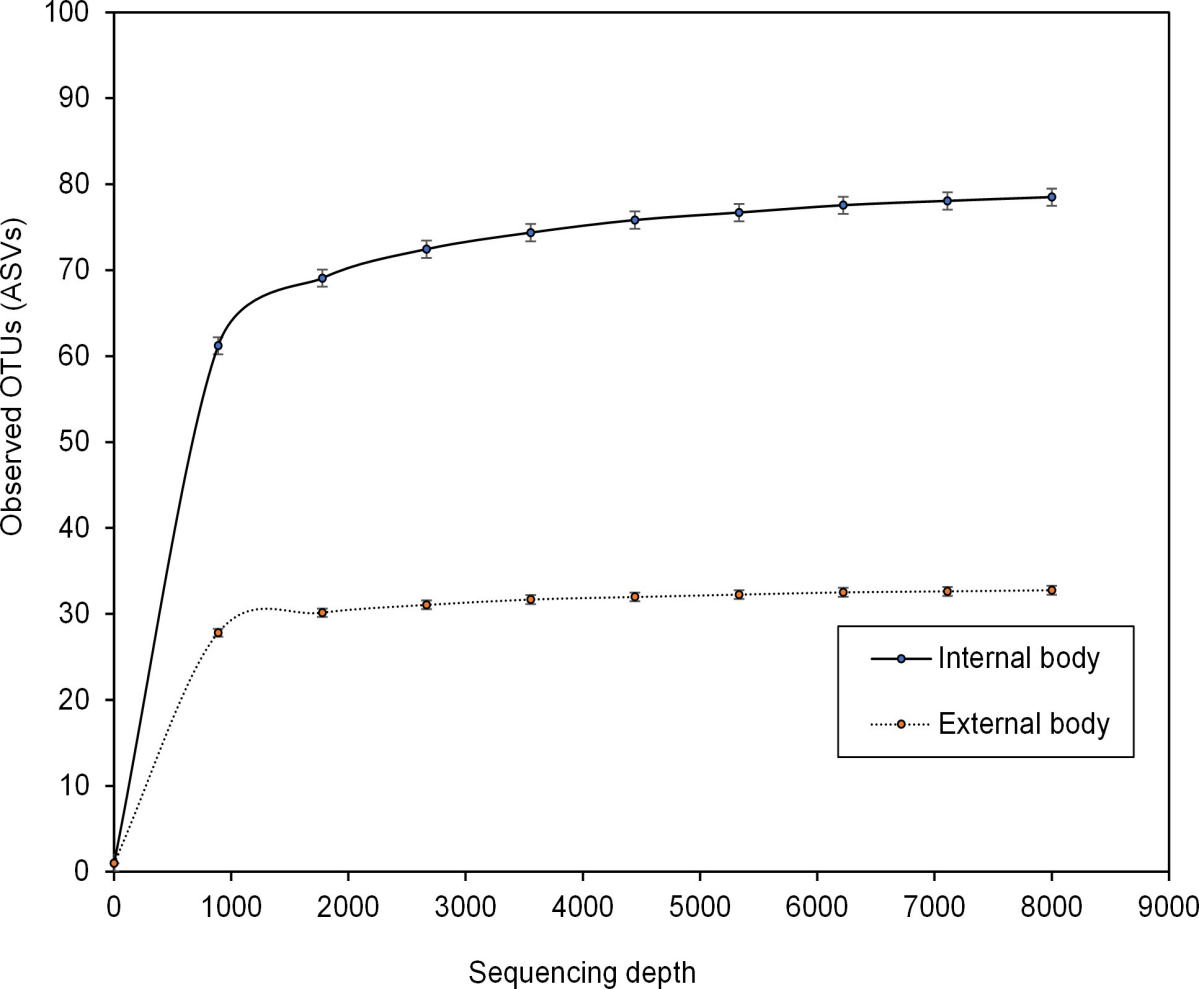
Rarefaction curves of the mean number of observed ASVs in internal versus external body samples.

**Fig 2:**
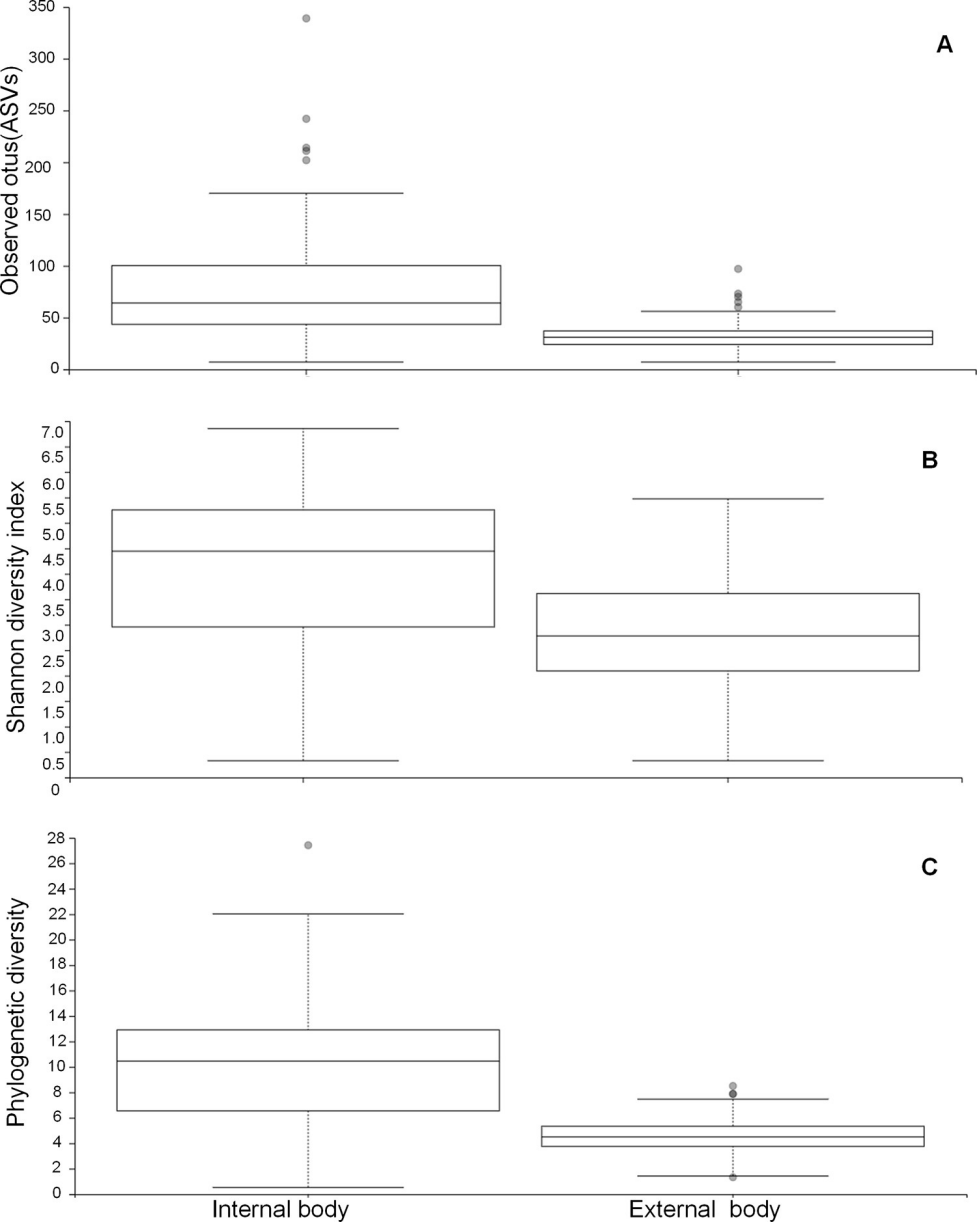
Alpha diversity measures of the internal and external body microbiomes of *Anopheles coluzzii* mosquitoes. (A) Observed OTUs, (B) Shannon diversity and (C) Faith’s phylogenetic diversity.

The rarefaction curves of the observed ASVs saturation in all the samples indicated that our sequencing depth was adequate to retrieve most of the taxa present in the samples (Fig 1, S2 Fig for all homes). The alpha diversities measuring the difference between the internal body and external surface wash samples differed significantly. The number of observed OTUs (Fig 2A) was significantly higher in the internal body than in the external surface wash samples (Kruskal-Wallis, p < 0.001). The Shannon index (Fig 2B), which takes into account both species richness and evenness, was also significantly higher in the inside (Kruskal-Wallis, p < 0.001). When taking the phylogenetic relationships into account, the Faith’s phylogenetic diversity (Fig 2C) was significantly higher (Kruskal-Wallis, p < 0.001) in the internal body samples. Between seven homes (internal body or external body samples), no significant differences (Kruskal-Wallis, p > 0.05) were observed (S3 and S4 Figs).

### Beta diversity

**Fig 3:**
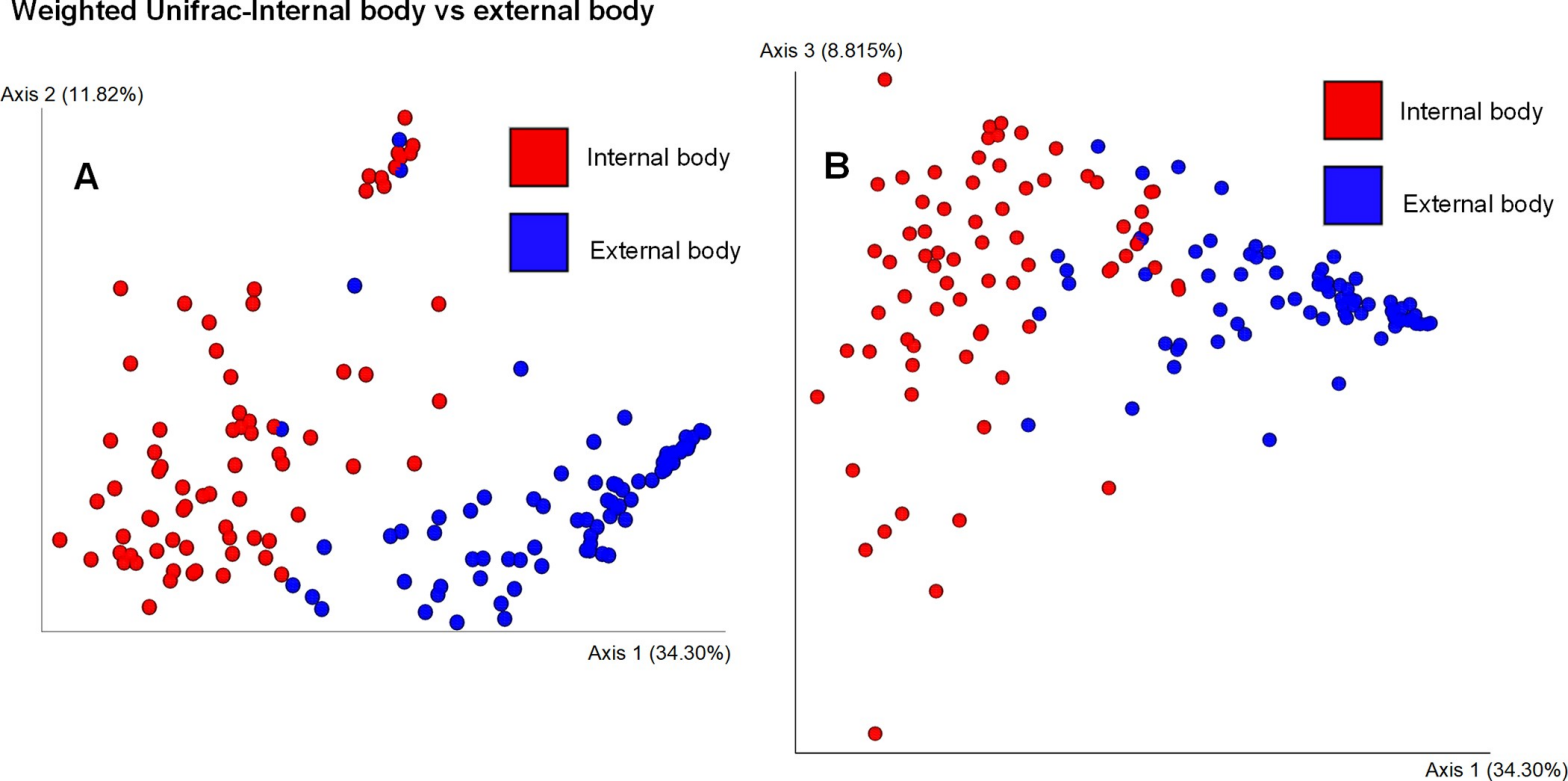
Principal coordinate analysis (PCoA) of bacterial composition between internal and external body samples of *Anopheles coluzzii* mosquitoes. Analysis was based on the weighted Unifrac metric.

**Fig 4:**
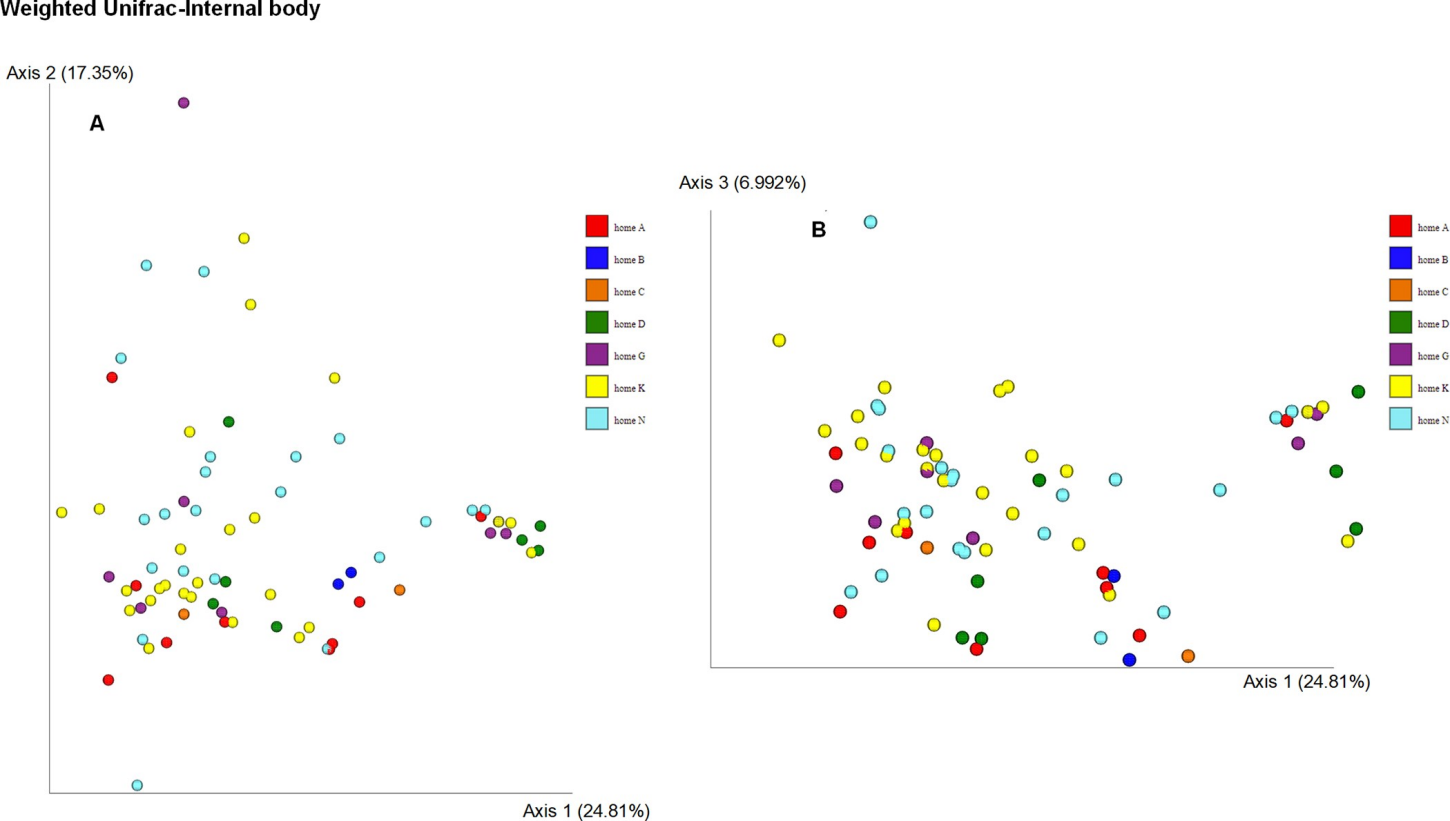
Principal coordinate analysis (PCoA) of the bacterial composition of the internal body of *Anopheles coluzzii* mosquitoes from seven homes. Analysis was based on the weighted Unifrac metric.

**Fig 5:**
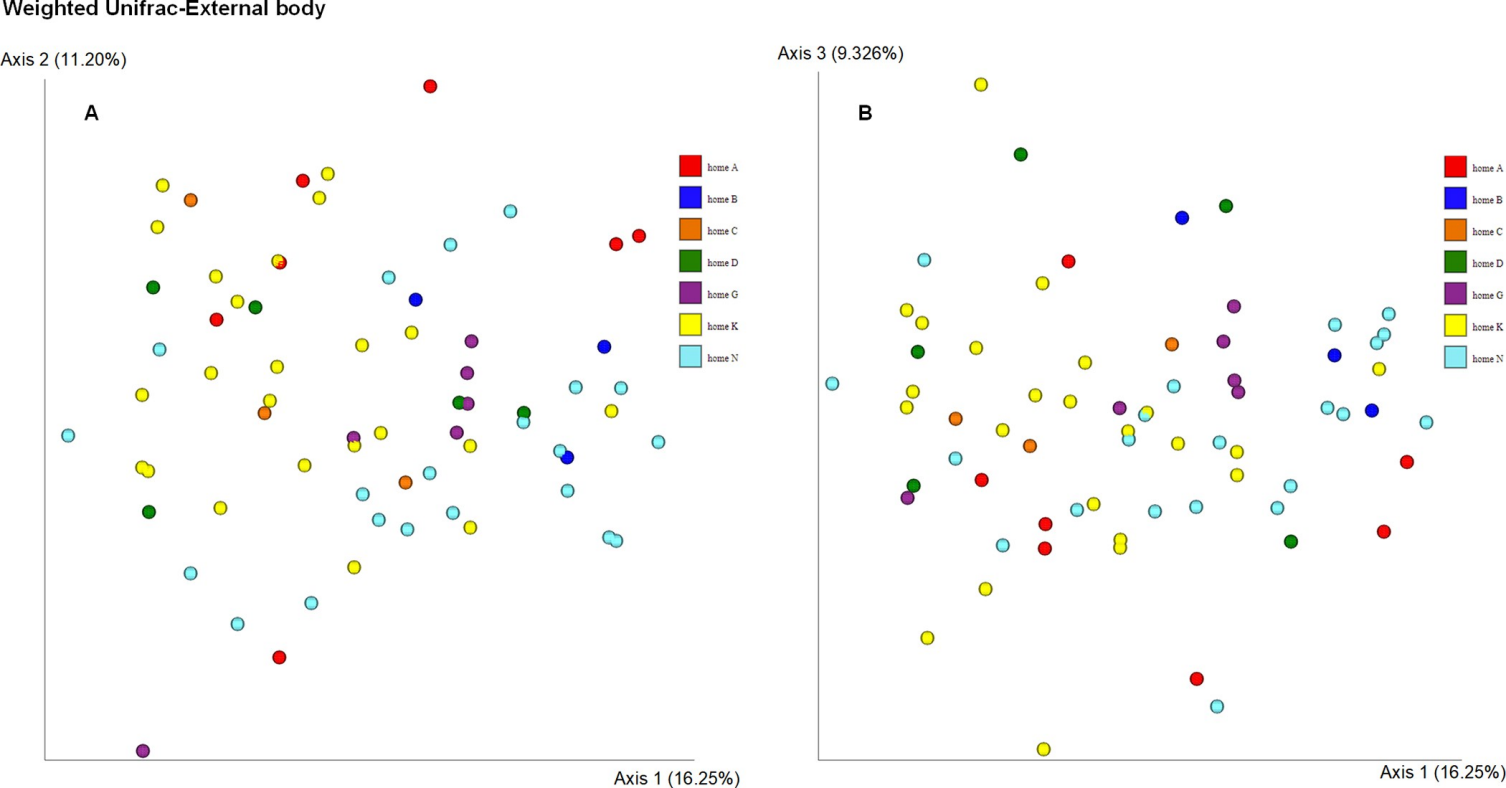
Principal coordinate analysis (PCoA) of the bacterial composition of the external body of *Anopheles coluzzii* mosquitoes from seven homes. Analysis was based on the weighted Unifrac metric.

Weighted Unifrac distance using Principal coordinates analysis (PCoA) (Fig 3) revealed two distinct clusters, the bacterial microbiota on the surface and the internal body of *Anopheles coluzzii* (PERMANOVA, p = 0.001). When analyzing the weighted Unifrac distances metric, the bacterial communities in the internal body samples from seven homes were similar (PERMANOVA, p = 0.11) (Fig 4). Significant differences were observed when comparing bacterial communities of external body samples from seven homes using the weighted Unifrac distance metric (PERMANOVA, p = 0.006) (Fig 5). Specifically, significant differences were observed between home A and home G (p = 0.037), home A and home K (p = 0.037), home A and home N (p = 0.037), and home K and home N (p = 0.021).

Non-phylogenetic distance metric Bray Curtis using Principal coordinates analysis (PCoA) (S5 Fig) also revealed two distinct clusters (PERMANOVA, p = 0.001). When analyzing the Bray Curtis distances metric, the bacterial communities in the internal body samples from seven homes were significantly different (PERMANOVA, p = 0.001) (S6 Fig). Significant differences were observed when comparing bacterial communities of external body samples from seven homes using the Bray Curtis distance metric (PERMANOVA, p = 0.001) (S7 Fig). In addition, we analyzed the data without the houses that have three specimens with both Unifrac and Bray Curtis. The results were basically the same; only with the Bray Curtis was there marginal non-statistical significance when the sample size per home was limited to >3.

### Bacterial community composition

**Fig 6:**
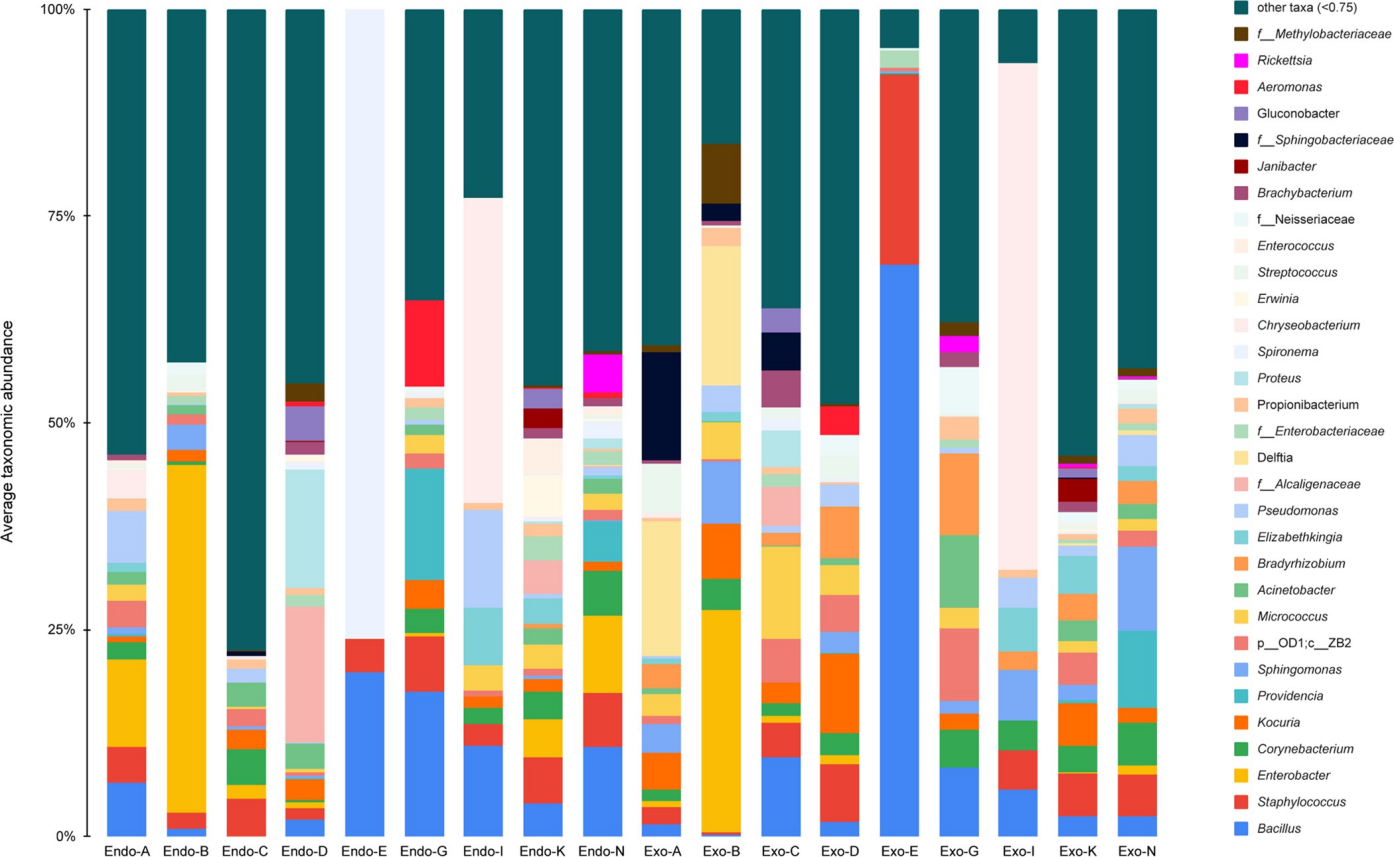
Relative abundances of major bacterial taxa at the genus level. ‘Other’ group represents all taxa with relative abundance below 0.75%. Each bar plot represents the mean sample composition for each home. Endo represents internal body samples, while Exo represents external body samples. Some taxa were not found at the genus level. “f_” represents family; “p_” represents phylum, and “c_” represents class.

At the phyla level of taxonomic analysis, four of the most common phyla found across all the samples were Proteobacteria, Firmicutes, Actinobacteria, and Bacteroidetes (S8 Fig). These four phyla accounted for 89.04% of all classified reads in all samples. At the order level, Actinomycetales, Bacillales, Enterobacteriales, Burkholderiales, Lactobacillales, Sphingomonadales, Pseudomonadales and Rhizobiales were predominant in all samples which accounted for 70.7% of all reads. At the family level, Enterobacteriaceae, Bacillaceae, Micrococcaceae, Staphylococcaceae, Sphingomonadaceae, and Corynebacteriaceae were found to be most abundant (S9 Fig). At the level of genus, the most abundant genera identified across all samples were *Bacillus* (5.6% of reads), *Staphylococcus* (4.8%), *Enterobacter* (4.4%), *Corynebacterium* (3.4%), *Kocuria* (2.8%), *Providencia* (2.7%) and *Sphingomonas* (2.5%) (Fig 6).

Internally, the predominant genera were *Bacillus* (7.3% of all internal body reads), *Enterobacter* (7.2%), *Staphylococcus* (5.2%), and *Corynebacter* (3.3%). Externally, the predominant genera were *Sphingomonas* (4.6% of all external body reads), *Staphylococcus* (4.5%), *Kocuria* (4.1%) and *Bacillus* (3.9%). The BLASTn results of the sequences for the taxa with relative abundance greater than 1% are shown in Table 1.

**Table 1:** BLASTn results of the sequences of the taxa with relative abundance higher than 1% in external body or internal body samples.

Taxa classified by Greengeens (External: internal abundance %)	Closest cultured bacteria/sequence from NCBI (strain)	Classification (Phylum)	Similarity (%)	Closest match NCBI accession #	Habitats
*Aeromonas* (0.37:1.26)	*Aeromonas hydrophila* (DSM 30187)	Proteobacteria	100	NR_119190	Fresh water and sewage; human pathogen-major bacterial foodborne diseases [99]
*Acinetobacter* (2.29: 1.83)	*Acinetobacter johnsonii* (ATCC 17909)	Proteobacteria	99.30	NR_117624	Skin [64]
*Alloiococcus* (1.00: 0.38)	*Dolosigranulum pigrum* (NBRC 15550)	Firmicutes	99.77	NR_113774	Human nasal microbiota [103]
*Bacillus* (3.92: 7.30)	*Bacillus cereus* (CCM 2010)	Firmicutes	99.77	MT421928	Commonly in the environment; GI syndrome [98]
*Corynebacterium* (3.54: 3.27)	*Corynebacterium casei* (LMG S-19264)	Actinobacteria	99.76	CP004350	Smear-ripened cheese [84]
*Dechloromonas* (0: 1.16)	*Azonexus caeni* (Slu-05)	Proteobacteria	99.30	NR_041017	Sludge of a wastewater treatment plant [89]
*Delftia* (2.64: 0.90)	*Delftia tsuruhatensis* (NBRC 16741)	Proteobacteria	99.77	NR_113870	Sludge of a wastewater treatment plant [90]
*Diaphorobacter* (1.05: 0)	*Diaphorobacter nitroreducens* (NA10B)	Proteobacteria	99.77	NR_024782	Sludge [88]
*Elizabethkingia* (2.22: 1.33)	*Elizabethkingia anophelis* (R26)	Bacteroidetes	99.76	CP023401	Midgut microbiota of mosquito [104]
*Enterobacter* (1.65: 7.16)	*Enterobacter xiangfangensis* (LMG27195)	Proteobacteria	100	CP017183	Chinese traditional sourdough [84]
*Enterococcus* (0.11: 1.70)	*Enterococcus raffinosus* (1789-79)	Firmicutes	99.77	NR_026499	Human blood culture [105]
*Erwinia* (0.84:1.80)	*Pantoea agglomerans* (FDAARGOS 1447)	Proteobacteria	100	CP077366	Eucalyptus leaves [80]
*Fructobacillus* (0.01: 1.30)	*Fructobacillus fructosus* (JCM 1119)	Firmicutes	100	LC062898	Fructose-rich niches [71]
*Gluconobacter* (0.50:1.15)	*Neokomagataea tanensis* (AH13)	Proteobacteria	99.75	CP032485	Flowers of candle bush [73]
*Kocuria* (4.06: 1.61)	*Kocuria rhizophila* (TA68)	Actinobacteria	99.51	NR_026452	Skin [67]
*Micrococcus* (2.30: 2.03)	*Micrococcus luteus* (ATCC 4698)	Actinobacteria	100	CP035298	Human skin flora [63]
*Propionibacterium* (1.21:1.00)	*Cutibacterium avidum* (ATCC 25577)	Actinobacteria	100	KF906606	Skin [66]
*Proteus* (0.33:1.75)	*Proteus mirabilis* (ATCC 29906)	Proteobacteria	100	NR_114419	Water, soil, and GI tracts of humans and animals [106]
*Providencia* (2.82: 2.63)	*Providencia rettgeri* (NCTC 11801)	Proteobacteria	99.77	NR_115880	soil and water;Travelers’ diarrhea [100]
*Pseudomonas* (1.99: 1.46)	*Pseudomonas alcaligenes* (NBRC 14159)	Proteobacteria	99.77	NR_113646	Swimming pool water [107]
*Rickettsia* (0.36: 1.26)	*Rickettsia bellii* (369L42-1)	Proteobacteria	99.75	NR_036774	Tick [93]
	*Rickettsia typhi* (Wilmington)	Proteobacteria	99.75	NR_074394	Flea [94]
*Sphingomonas* (4.61: 0.42)	*Sphingomonas paucimobilis* (FDAARGOS_908)	Proteobacteria	99.75	CP065670	Soil and water [108]
*Spironema* (0.14:1.84)	*Alkalispirochaeta cellulosivorans* (JC227)	Spirochaetes	83.26	NR_148863	Gut of a wood-eating cockroach [109]
*Staphylococcus* (4.48: 5.18)	*Staphylococcus haemolyticus* (ATCC 29970)	Firmicutes	100	KT989857	Skin [65]
*Streptococcus* (2.21: 0.32)	*Streptococcus dysgalactiae* (FDAARGOS_1157)	Firmicutes	100	CP068057	Bovine pathogen [110]
*Swaminathania* (0.09:1.03)	*Asaia bogorensis* (NBRC 16594)	Proteobacteria	99.75	AP014690	Flowers of the orchid tree (*Bauhinia purpurea*) [74]
*Thorsellia* (0.15:1.01)	*Thorsellia anophelis* (CCUG 49520)	Proteobacteria	99.77	AY837748	Midgut of the malaria mosquito *Anopheles arabiensis* [111]

Significant differences (t-tests, α = 0.05) in relative abundance for *Enterobacter*, *Kocuria*, *Sphingomonas*, ZB2, *Bradyrhizobium*, and Enterobacteriaceae were found between internal body and external body samples (Table 2). *Enterobacter* and Enterobacteriaceae were significantly abundant in the internal body sample, while *Kocuria*, *Sphingomonas* and *Bradyrhizobium* were significantly abundant in the external body samples (Table 2). *Rickettsia* sequences were detected in internal body samples N23 (95.3%, see S2 File), G6 (0.18%) and A5 (0.004%), and in external body samples G5s (13.5%), K9s (10.8%) and N23s (3.6%).

**Table 2:** Differences in the abundance of common microbiota taxa in internal body and external body samples.

Taxa	Average (± SE) abundance (%)		*df*	*p*
	Internal body (n = 76)	External body (n = 77)		
*Bacillus*	7.30 (1.76)	3.92 (1.26)	136.26	0.0605
*Staphylococcus*	5.18 (0.84)	4.48 (0.75)	148.65	0.2678
*Enterobacter*	7.16 (2.23)	1.65 (1.04)	106.22	0.0137*
*Corynebacterium*	3.27 (0.67)	3.54 (0.71)	150.69	0.3888
*Kocuria*	1.61 (0.49)	4.06 (1.06)	106.58	0.0193*
*Providencia*	2.63 (1.68)	2.82 (1.52)	149.26	0.4674
*Sphingomonas*	0.42 (0.16)	4.61 (1.32)	78.31	0.0011*
p__OD1;c__ZB2	1.31 (0.23)	3.33 (0.59)	98.59	0.0009*
*Micrococcus*	2.03 (0.40)	2.30 (0.65)	127.57	0.3658
*Acinetobacter*	1.83 (0.40)	2.29 (0.54)	139.40	0.2470
*Bradyrhizobium*	0.20 (0.10)	3.66 (0.94)	77.66	0.0002*
*Elizabethkingia*	1.33 (0.51)	2.22 (0.83)	125.41	0.1808
*Pseudomonas*	1.46 (0.43)	1.99 (0.53)	144.93	0.2178
f__Alcaligenaceae	2.88 (1.65)	0.18 (0.18)	76.84	0.0547
*Delftia*	0.90 (0.04)	2.64 (0.96)	76.32	0.0050
f__Enterobacteriaceae	1.71 (0.45)	0.55 (0.23)	112.79	0.0123*
*Propionibacterium*	1.00 (0.17)	1.21 (0.38)	104.83	0.3077
*Proteus*	1.75 (1.35)	0.33 (0.23)	79.46	0.1519

### Differential abundance

The ANCOM test showed that two ASVs from Sphingobacteriaceae and one ASV from *Delftia* significantly differed among different homes in the external body samples (S10 Fig). In 75% of the samples in home C, D, K, and N, one or fewer sequences were observed to have been assigned to *Delftia* (S2 Table). However, in 75% of the samples in home A, 4550.75 or fewer sequences were assigned to *Delftia*, and in 75% of the samples in home B, 6136.5 or fewer sequences were assigned to *Delftia*. The genus *Delftia* was higher in homes A and B than in homes C, D, K, and N among all external body samples. The ANCOM percentile abundance also suggested that the two ASVs from Sphingobacteriaceae were higher in home A than in other homes. In 75% of the internal body samples in homes B, C, D, K, and N, one or fewer sequences were observed to have been assigned to *Dechloromonas* (S2 Table). However, in 75% of the samples in home A, 17395 or fewer sequences were assigned to *Dechloromonas*. This suggests that *Dechloromonas* was higher in home A than in other homes among all internal body samples.

### *Rickettsia* phylogenetic analyses

**Fig 7:**
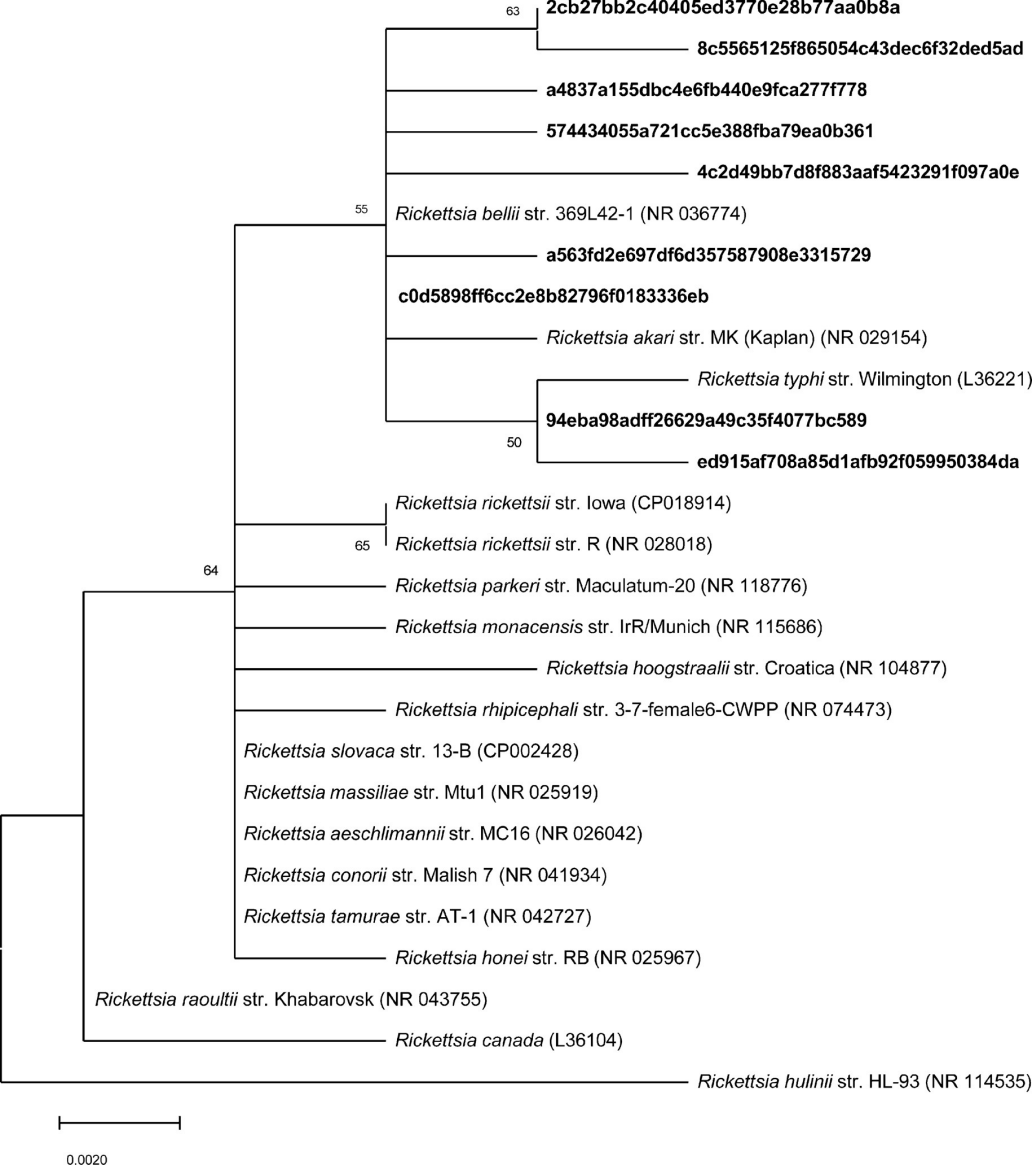
Molecular phylogenetic analysis of *Rickettsia* amplicon sequence variants (ASVs) identified in this study (in bold text) and closest related species. Phylogenetic tree constructed by the maximum likelihood method based on 16S rRNA gene. The percentage of replicate trees with 50% cutoff value in which the associated taxa clustered together in the bootstrap test (1000 replicates) are shown below the branches.

To further identify *Rickettsia* spp. sequences, nine ASVs that were identified in the genus *Rickettsia*, and 18 closest related species from BLASTn searches were used to construct phylogenetic trees based on the maximum-likelihood (Fig 7) and neighbor-joining (S11 Fig). The phylogenetic analysis suggests that seven of the *Rickettsia* ASVs clustered to *Rickettsia bellii* str. 369L42-1 (NR036774) with an over 99.99% similarity; two of the ASVs clustered to *Rickettsia typhi* str. Wilmington (L36221) with an over 99.99% similarity.

## Discussion

In this study, we investigated the bacterial community of the internal body versus external body surfaces of female *An*. *coluzzii* adults collected from homes in the West African country of Côte d’Ivoire, Africa. Our results revealed that *An*. *coluzzii* harbor a diverse microbiota, likely influenced by mosquito development, its ecology inside and outside of homes, and how mosquitoes feed. Alpha diversity metrics, the number of observed OTUs, Shannon diversity, and Faith’s phylogenetic diversity were all significantly higher in the internal body samples than outside of the insect. The principal coordinates analysis (PCoA) of the beta diversity and weighted unifrac metrics showed two clusters of samples, suggesting that the bacterial microbiota on the surface and internal to the body of *An*. *coluzzii* were significantly different. Similar comparisons in other insects are limited. Deguenon et al. [6] compared the internal and external body microbiota of blow flies trapped on an urban farm on our NC State University campus. Park et al. [53] conducted similar work for house flies collected from a farm, home, hospital, and laboratory colony. These two studies found the external body bacterial population was more diverse than the internal population in most cases, which was contrary to our work here with mosquitoes [53]. Exceptions for the house fly research of Park et al. [53] were observed for insects obtained from a hospital and laboratory colony, where no significant differences were found based on alpha diversity; the mean value of the Shannon diversity and observed ASVs were higher internally than externally for the laboratory house flies.

Our working hypothesis based on earlier studies with blow flies [6] was that bacterial diversity would be greater on the outside compared to the inside. Bacteria found inside have to be adapted to the internal structure and physiology of the insect and resist its natural immunity. Bacteria on the outside are mostly a function of physical interactions with surroundings. Furthermore, blow flies and house flies, might have greater bacterial diversity on their surface than mosquitoes for several reasons. For example, *Anopheles* larvae live mostly in rice paddies, edges of rivers, and streams in the rainy season of Côte d’Ivoire [54], while filth fly larvae live in bacteria-rich environments, e.g., garbage, waste and manure and are adapted to a saprophagous diet [55]. Also, there are significant differences in the external morphology between filth flies and mosquitoes that might favor the mechanical transfer of bacteria to the surface of filth flies. For example, setae size and density and tarsal pads are different between filth flies and mosquitoes. The longer legs and resting behavior of mosquitoes also might reduce the mosquito body from touching surfaces. Blow flies and house flies are stronger fliers than mosquitoes, they interact with different substrates in their habitat with a greater shear force than mosquitoes and move more frequently and at greater distances [56, 57].

Mosquitoes differ from filth flies because they feed on blood (which is essentially sterile). The mosquitoes in our study were collected inside of homes early in the morning. Based on visual inspection at the time of mosquito homogenization, it appears the insects did not contain blood (no appearance of red color). Mosquitoes immediately after blood feeding become quiescent within a short distance from where they obtained their blood meal and during this quiescent period, they digest the blood meal; our collection method would favor the collection of these mosquitoes. Gusmão et al. [58] and Wang et al. [14] reported that bacterial abundance in *An*. *gambiae* and *Aedes aegypti* increased dramatically after blood feeding. The bacterial load peaked at 48h post blood-feeding, with high levels in the posterior midgut, peritrophic matrix, and around the blood meal, suggesting that bacteria are either benefiting from the nutrients released in these digestive areas and maybe helping to digest blood [58]. Sharma et al. [59] found that the salivary gland of *Anopheles culicifacies* harbor a more diverse microbial community than the digestive system. Our findings of a greater bacteria diversity inside of the mosquito is likely resulting from a combination of factors including differences in habitat, morphology, behavior and feeding differences from filth flies, where bacteria diversity was greater on the outside. More research is needed to obtain a better understanding of this question.

We also observed significant differences in the external body microbiota between homes which was not the case for the internal body samples. These results support the hypothesis that internal bacteria diversity is less affected by environmental exposure compared to what is found on the outside of mosquitoes. Mosquitoes generally are not considered to be filth flies, but at least the potential is there that when they migrate into homes seeking a blood meal, they could also be transferring bacteria from their habitat outside of homes. This will be discussed in more detail later. Mosquitoes also are likely acquiring different bacteria specific to the home. Dunn et al. [60] sampled nine distinct locations in forty homes in Raleigh and Durham, North Carolina, US and found significant bacteria variability between homes. The surface materials, human and animal occupants, the size and cleaning frequency of the home, and indoor humidity and temperature are attributed to this bacterial diversity across homes [60]. This could explain the variability in the bacteria microbiota on the surface of mosquitoes between homes in our study. It is not surprising that the outside surface bacteria microbiota is affected by the mosquito habitat and also suggests that mosquitoes could potentially mechanically transfer human and animal pathogens. The latter has been understudied.

Proteobacteria, Firmicutes, Actinobacteria and Bacteroidetes (from highest to lowest abundance) were the most dominant phyla both internally and externally in our mosquito samples from homes in Côte d’Ivoire. In internal microbiome studies of other dipterans, the order of the latter three phyla varied. However, Proteobacteria was always the most predominant phylum. This was the case for the *An*. *gambiae* complex mosquito midgut microbiome in Burkina Faso [21], eight *Anopheles* species in Thailand [25], house flies [61], blow flies [6], and fruit flies [62].

BLAST searches were conducted for all bacterial genera that accounted for more than 1% of the internal or external body reads. We identified *Staphylococcus haemolyticus* (external vs internal abundance 4.48%: 5.18%, respectively), *Kocuria rhizophila* (4.06%: 1.61%), *Acinetobacter johnsonii* (2.29%: 1.83%), *Micrococcus luteus* (2.30%: 2.03%), and *Cutibacterium avidum* (1.21%: 1.00%) found on human skin [63–67]. *Staphylococcus haemolyticus* is involved in nosocomial infections, is the second most common coagulase-negative *Staphylococcus* isolated from clinical sites [68], and is also known to be multidrug resistant [69]. Interestingly, a recent study showed that *Staphylococcus* was found to be more abundant among skin microbiota of women who are highly attractive to *An*. *coluzzii* than poorly attractive group [70]. It is possible that the bacterial genera *Staphylococcus* detected in this study could be acquired by female mosquitoes when feeding.

In this study, several plant-associated bacteria species of *Fructobacillus fructosus* (external vs internal abundance--0.01%:1.30%, respectively), *Neokomagataea tanensis*(0.50%:1.15%), *Asaia bogorensis* (0.09%:1.03%) and *Pantoea agglomerans* (0.84%:1.80%) were identified and were more abundant inside versus outside of our mosquitoes. This is the first identification of a *Fructobacillus* species inside of a mosquito. *Fructobacillus fructosus* is a member of the fructophilic lactic acid bacteria group found in fructose-rich niches [71], for example, in flowers, fruits [72], honeybees and beehives. Plant nectar is an essential source of female mosquito nutrition. Female mosquito immediate mortality due to sugar deprivation does not occur but does reduce fecundity potentially from the reduced production of juvenile hormone [33]. *Neokomagataea tanensis* was first isolated from lantana flowers and candle bush in Thailand in 2011 [73]. The acetic acid bacterium, *Asaia bogorensis*, was first isolated from the flower nectar of the orchid tree, *Bauhinia purpurea* [74]. This bacteria was subsequently found in *Anopheles* mosquitoes and reported to impact larval development [75]. The discovery of the natural Asaia/*Anopheles* mosquitoes/flower nectar cycle was recently considered a possible delivery tool for *Asaia*-based paratransgenetic malaria control [76–78]. *Asaia* was also reported to likely have an indirect role in reducing the vectorial capacity of *Anopheles* mosquitoes by inhibiting the *Plasmodium* sporogenic cycle [76]. In other studies, the genus *Asaia* was reported to be dominated in deltamethrin insecticide susceptible *An*. *coluzzii* collected from Agboville, Côte d’Ivoire [79]. Our findings of these three bacteria associated with plant nectar suggest the *An*. *coluzzii* mosquitoes in our samples visited plants for nectar before entering the homes. *Pantoea agglomerans* also are associated with plants [80] and found associated with honey bees [81], aphids, and mosquitoes [82]. Riehle et al. [83] suggested *P*. *agglomerans* was a good candidate for paratransgenic control of *Plasmodium*.

Two bacteria species, *Corynebacterium casei* and *Enterobacter xiangfangensis*, typically associated with fermented food were identified in our mosquito samples. *C*. *casei* were found at approximately the same levels external vs internal (3.54%:3.27%, respectively). *En*. *xiangfangensis* (1.65%:7.16%) were found significantly higher internally. *C*. *casei* and *En*. *xiangfangensis* were reported before in smear-ripened cheese [84] and traditional Chinese sourdough [85], respectively. Peach et al. [86] reported that host-seeking mosquitoes were strongly attracted to homemade cheese. *An*. *gambiae* also is attracted to sugar-fermenting yeast because of the carbon dioxide produced by yeast [87]. *C*. *casei* and *En*. *xiangfangensis* identified in our samples, suggested the *An*. *coluzzii* mosquitoes might have visited fermented foods in the homes where they were found.

Three bacteria species, *Delftia tsuruhatensis* (external vs internal abundance--2.64%: 0.90%, respectively), *Diaphorobacter nitroreducens* (1.05%:0%) and *Azonexus caeni* (0%:1.16%) are associated with aquatic habits where mosquito larvae live. These three bacteria were all isolated from treatment plant wastewater sludge [88–90]. Zogo et al. [54] reported *Anopheles* larvae live mostly in rice paddies in Côte d’Ivoire. Rice paddies are flooded with water producing anaerobic conditions similar to sludge in wastewater. *Delftia* was found both internally and externally and was identified higher in homes A and B than in the other homes among all external body samples (S10 Fig). The relative abundance of *Delftia* in external body samples versus internal samples was 55.7% and 0.4%, respectively; for home B, 35.3% of the reads were identified as *Delftia* in external body samples while no reads were found in the internal samples (see S2 File). This suggests that mosquitoes in homes A and B have been to *Delfti*a rich environments or the larval stages have acquired *Delftia* by feeding and transmitted transstadially to adults. *Diaphorobacter* was only found in external samples. *Azonexus* was found in internal samples and was only detected in samples in home A. This suggests the larval habit of mosquitoes in home A contained this unique bacterium. It is possible that *Azonexus* was transferred transstadially from the mosquito larvae to the adult. The other possibility is the bacteria was acquired from the larval habitat from the consumption of water after emerging from the pupa.

*Rickettsia* sequences were detected in three internal sample, N23 (relative abundance- 95.3%), G6 (0.18%) and A5 (0.004%) and three external samples, G5s (13.5%), K9s (10.8%) and N23s (3.6%). Even though the number of samples detected with *Rickettsia* sequences were the same, the number of reads in the internal samples (147,884 reads) were more than in the external samples (5,244 reads). It is surprising that *Rickettsia* spp. sequences were detected outside of the body since they are intracellular bacteria. The mosquitoes were intact when washing was conducted with PBS buffer to obtain the outside bacteria community. It is possible that pieces of leg or setae or maybe internal content from a severed body part or the rectum separated from the rest of the insect during washing which was not noticed. Further, no obvious defecation was noticed, but it is theoretically possible that gut contents could leak out through the anus even for an insect that is dead. This seems unlikely, especially considering the Illumina sequencing protocol is not nearly as sensitive as PCR, nested PCR in detecting specific bacteria DNA, and requires a robust level of bacterial genomic DNA to detect bacteria. It is also possible that the *Rickettsia* spp. sequences were from ectoparasitic mites that parasitize mosquitoes [91]. It was shown before that 36.45% of *Anopheles* mosquitoes were parasitized with mites [91]. We did not anticipate this possibility and did not search for mites on our mosquito samples.

The closest match of *Rickettsia spp*. sequences using BLAST searches were *Rickettsia bellii* 369L42-1 with a 99.75% similarity (NR_036774) and *Rickettsia typhi* Wilmington with a 99.75% similarity (NR_074394). *Rickettsia bellii* was found in mosquitoes in China, and 2.3% (70/3051) of the sampled mosquitoes, including one *Anopheles* species, were infected with *R*. *bellii* [92]. *Rickettsia bellii* was widely distributed in multiple tick species in the United States [93]. *Rickettsia bellii* is the ancestral group-*Rickettsia* species, and it could play a crucial role in the ecology and epidemiology of other pathogenic tick-borne spotted fever group *rickettsiae* [93]. *Rickettsia typhi* is the causative agent of murine typhus, also known as endemic typhus [94]. *Rickettsia typhi* can be transmitted to humans by bites and feces of infected fleas. Infected people can have symptoms like a fever, rash and bronchitis and produce severe disease in vulnerable populations like the elderly and immune-compromised people. If left untreated, murine typhus can be fatal, with a 4% death rate [95]. *Rickettsia typhi* was not reported before in mosquitoes. However, *R*. *felis*, reported to be the possible causative agent of murine typhus [96], was found in *An*. *gambiae* from Sub-Saharan Africa [97].

Noteworthy, several food borne pathogenic bacteria were also found both externally and internally. *Bacillus cereus* were more abundant among all bacteria in the internal samples (3.92%:7.30%, respectively). Of the mosquitoes collected, *B*. *cereus* was found in 67.1% (51/76) of the internal samples and 31.2% (24/77) in the external samples (see S2 File). *Aeromonas hydrophila* (0.37%:1.26%) were more abundant in the internal samples and found in 22.4% (17/76) of the internal samples of the mosquitoes collected and 2.6% (2/77) for the external samples (see S2 File). *Bacillus cereus* is a pathogen associated with food poisoning, diarrhea and other gastrointestinal disorders [98]. *Aeromonas hydrophila* is frequently found in fresh water and sewage and can cause *Aeromonas* enteritis and is a major bacterial foodborne disease [99]. *Providencia rettgeri* (2.82%: 2.63%) was found both internally and externally. *Providencia rettgeri* is associated with traveller’s diarrhea [100,101]. Recent studies found *P*. *rettgeri* in stool samples from diarrhea patients and also in meat samples, suggesting *Providencia* infection in humans could come through meat [102]. These results suggest that they coud be transferred to surfaces in the home by both mechanical interations and defecation and potentially cause food borne diseases to humans. The role of mosquitoes as a filth fly is an understudied area of science and needs more research to assess their risk to humans.

## Conclusions

Our study provides the first study of bacterial communities from the internal body and external body of home-caught mosquitoes, i.e., *An*. *coluzzii*. The mosquitoes had a greater diversity of bacterial taxa internally than externally. The internal bacterial communities were similar between homes, while the external body samples were significantly different between homes. The bacteria on the external body were associated with plants, human and animal skin, and human infections. To our knowledge, Fructobacillus was identified in the internal body of mosquitoes for the first time. Internally, *R*. *bellii* and *R*. *typhi* were found, potentially of importance since this genus is associated with human and animal diseases. Based on these findings, further research is warranted to assess the potential mechanical transmission of bacteria by mosquitoes into homes as they seek a blood meal and the importance of their internal microbiota in human health.

## Supporting information

S1 Table Bacterial mock community expected from ZymoBIOMICS™ Microbial Community DNA and actual taxonomic abundances in our samples. (DOCX)

S2 Table Significantly differing taxa from ANCOM differential abundance testing resulted and the percentile abundance of taxa by group. (DOCX)

S1 Fig Map of areas where mosquitoes sampling was conducted house-by-house in a five-block area of Tiassalé, Côte d’Ivoire, Africa (5°53’54" N, 4°49’42" W). (TIF)

S2 Fig Rarefaction curves of the mean number of observed ASVs from seven homes. (TIF)

S3 Fig Alpha diversity measures of the internal body microbiomes of *Anopheles coluzzii* mosquitoes from seven homes. (A) Observed OTUs, (B) Shannon diversity and (C) Faith’s phylogenetic diversity.
(TIF)

S4 Fig Alpha diversity measures of the external body microbiomes of *Anopheles coluzzii* mosquitoes from seven homes. (A) Observed OTUs, (B) Shannon diversity and (C) Faith’s phylogenetic diversity.
(TIF)

S5 Fig Principal coordinate analysis (PCoA) of bacterial composition between internal and external body samples of *Anopheles coluzzii* mosquitoes. Analysis was based on the Bray Curtis metric.
(TIF)

S6 Fig Principal coordinate analysis (PCoA) of the bacterial composition of the internal body of *Anopheles coluzzii* mosquitoes from seven homes. Analysis was based on the Bray Curtis metric.
(TIF)

S7 Fig Principal coordinate analysis (PCoA) of the bacterial composition of the external body of *Anopheles coluzzii* mosquitoes from seven homes. Analysis was based on the Bray Curtis metric.
(TIF)

S8 Fig Relative abundances of major bacterial taxa at the phylum level. ‘Other’ group represents all taxa with relative abundance below 5%.
(TIFF)

S9 Fig Relative abundances of major bacterial taxa at the family level. ‘Other’ group represents all taxa with relative abundance below 0.75%.
(TIF)

S10 Fig ANCOM differential abundance testing result for (A) external body and (B) internal body. Significantly differing taxa from ANCOM differential abundance testing resulted and the percentile abundance of taxa by group are shown in S2 Table. QIIME2 was used for ANCOM analysis.
(TIF)

S11 Fig Molecular phylogenetic analysis of *Rickettsia* amplicon sequence variants (ASVs) identified in this study (in bold text) and closest related species. Phylogenetic tree constructed by the neighbor-joining method based on 16S rRNA gene. The percentage of replicate trees with 50% cutoff value in which the associated taxa clustered together in the bootstrap test (1000 replicates) are shown below the branches.
(TIFF)

S1 File Mapping file. (XLSX)

S2 File Data file. (XLSX)
