# Supplementary material for: Internal and external microbiota of home-caught Anopheles coluzzii (Diptera: Culicidae) from Côte d’Ivoire, Africa: Mosquitoes are filthy
Source: PLoS One. 2022 Dec 15;17(12):e0278912. doi: 10.1371/journal.pone.0278912 (PMC9754230; doi:10.1371/journal.pone.0278912)
Supplement: S1 Table — (DOCX) [file pone.0278912.s001.docx]

**S1 Table. Bacterial mock community expected from ZymoBIOMICS™ Microbial Community DNA and actual taxonomic abundances in our samples.**

| **Species** | **Theoretical composition (%)** | **Species** | **Actual composition (%)** |
| --- | --- | --- | --- |
| *Pseudomonas aeruginosa* | 4.2 | Pseudomonadaceae | 5.2 |
| *Escherichia coli* | 10.1 | Enterobacteriaceae | 8.8 |
| *Salmonella enterica* | 10.4 | *Salmonella enterica; Salmonella* | 22.9 |
| *Lactobacillus fermentum* | 18.4 | *Lactobacillus* | 21.9 |
| *Enterococcus faecalis* | 9.9 | *Enterococcus* | 3.1 |
| *Staphylococcus aureus* | 15.5 | *Staphylococcus* | 8.8 |
| *Listeria monocytogenes* | 14.1 | *Listeria* | 7.8 |
| *Bacillus subtilis* | 17.4 | *Bacillus* | 21.0 |
| *Methylobacterium* | 0 | *Methylobacterium* | 0.4 |
