# Supplementary material for: Internal and external microbiota of home-caught Anopheles coluzzii (Diptera: Culicidae) from Côte d’Ivoire, Africa: Mosquitoes are filthy
Source: PLoS One. 2022 Dec 15;17(12):e0278912. doi: 10.1371/journal.pone.0278912 (PMC9754230; doi:10.1371/journal.pone.0278912)
Supplement: S2 Table — (DOCX) [file pone.0278912.s002.docx]

**S2 Table. Significantly differing taxa from ANCOM differential abundance testing resulted and the percentile abundance of taxa by group.**

| **Home** | **Percentile** | **External body** | | | **Internal body** |
| --- | --- | --- | --- | --- | --- |
|  |  | **Sphingobacteriaceae-1** | ***Delftia*** | **Sphingobacteriaceae-2** | ***Dechloromonas*** |
| home A | 0 | 1 | 1 | 1 | 1 |
|  | 25 | 309.25 | 1261.25 | 1 | 1 |
|  | 50 | 389 | 3563.5 | 233 | 1904 |
|  | 75 | 2242.75 | 4550.75 | 1689.5 | 17395 |
|  | 100 | 4800 | 6536 | 6216 | 34566 |
| home B | 0 | 1 | 1033 | 221 | 1 |
|  | 25 | 1 | 3328.5 | 595.5 | 1 |
|  | 50 | 1 | 5624 | 970 | 1 |
|  | 75 | 1 | 6136.5 | 1077 | 1 |
|  | 100 | 1 | 6649 | 1184 | 1 |
| home C | 0 | 1 | 1 | 1 | 1 |
|  | 25 | 1 | 1 | 1 | 1 |
|  | 50 | 1 | 1 | 1 | 1 |
|  | 75 | 165 | 1 | 504.5 | 1 |
|  | 100 | 329 | 1 | 1008 | 1 |
| home D | 0 | 1 | 1 | 1 | 1 |
|  | 25 | 1 | 1 | 1 | 1 |
|  | 50 | 1 | 1 | 1 | 1 |
|  | 75 | 1 | 1 | 1 | 1 |
|  | 100 | 1 | 1 | 3 | 1 |
| home G | 0 | 1 | 1 | 1 | 1 |
|  | 25 | 1 | 1 | 1 | 1 |
|  | 50 | 1 | 1 | 1 | 1 |
|  | 75 | 1 | 1 | 1 | 1 |
|  | 100 | 1 | 1 | 1 | 1 |
| home K | 0 | 1 | 1 | 1 | 1 |
|  | 25 | 1 | 1 | 1 | 1 |
|  | 50 | 1 | 1 | 1 | 1 |
|  | 75 | 1 | 1 | 1 | 1 |
|  | 100 | 1 | 2748 | 1 | 1 |
| home N | 0 | 1 | 1 | 1 | 1 |
|  | 25 | 1 | 1 | 1 | 1 |
|  | 50 | 1 | 1 | 1 | 1 |
|  | 75 | 1 | 1 | 1 | 1 |
|  | 100 | 1 | 5480 | 1 | 1 |
