# Supplementary figures and images for: Internal and external microbiota of home-caught Anopheles coluzzii (Diptera: Culicidae) from Côte d’Ivoire, Africa: Mosquitoes are filthy
Source: PLoS One. 2022 Dec 15;17(12):e0278912. doi: 10.1371/journal.pone.0278912 (PMC9754230; doi:10.1371/journal.pone.0278912)

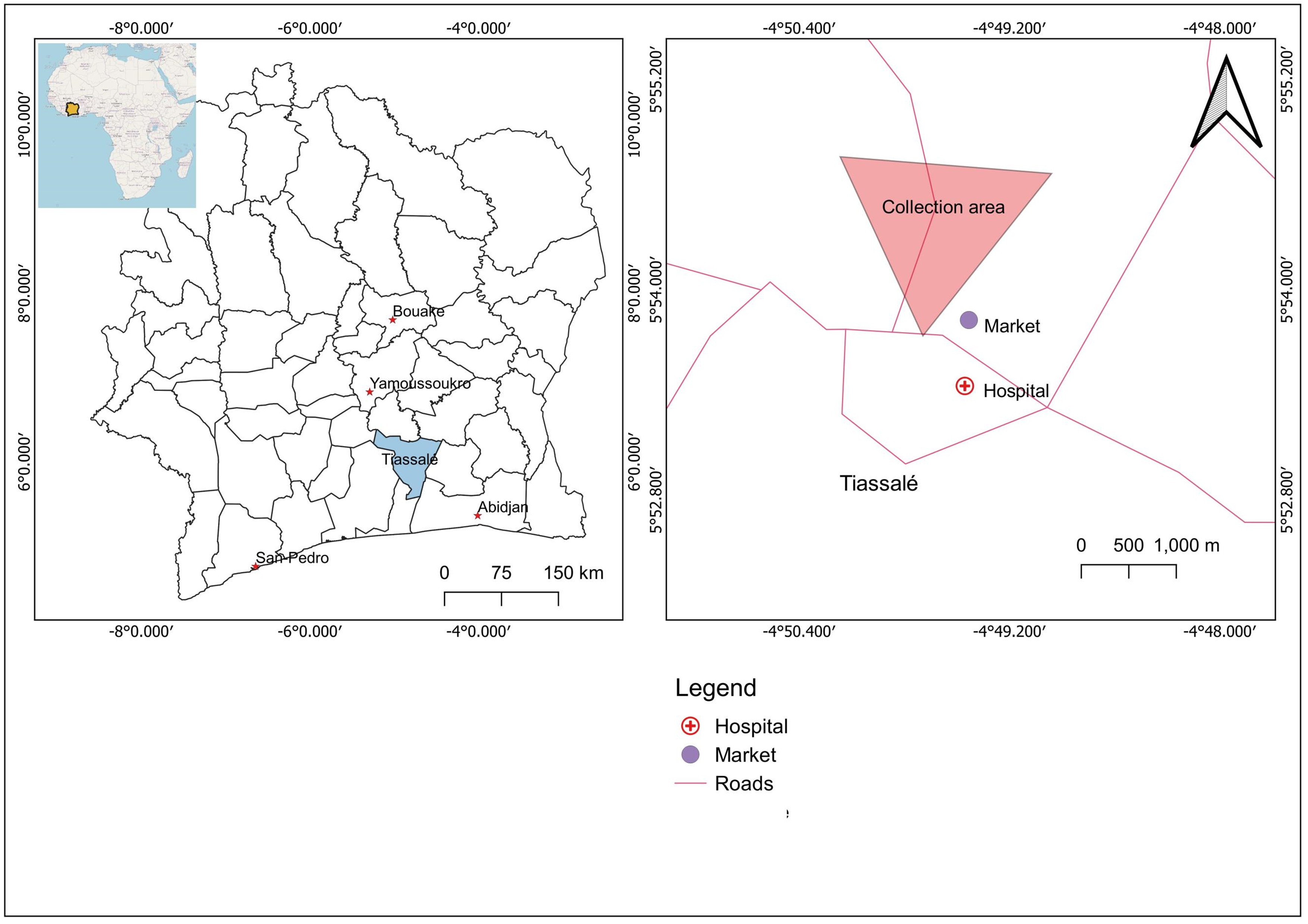

Supplement: S1 Fig — (TIF) [file pone.0278912.s003.tif]

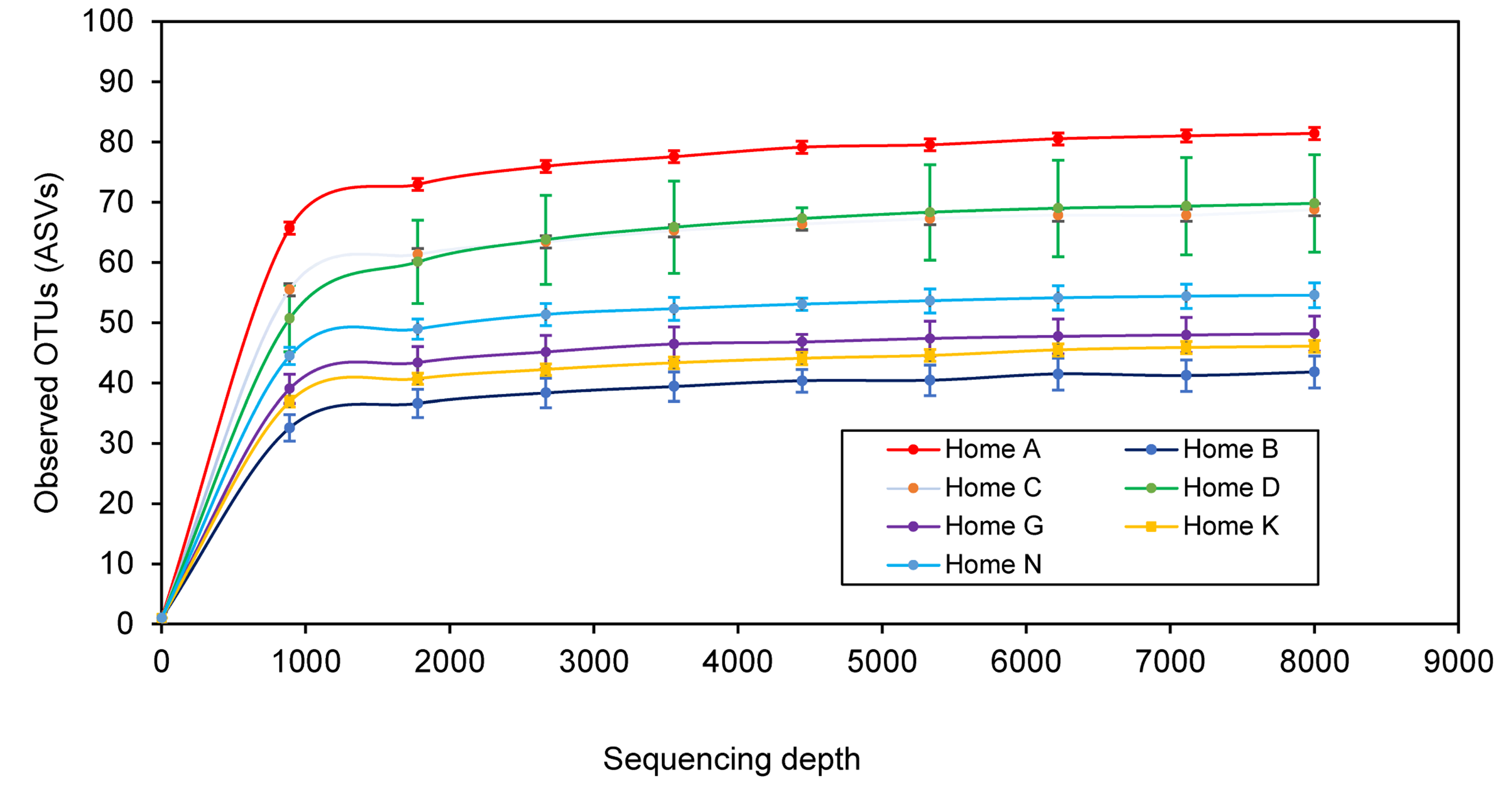

Supplement: S2 Fig — (TIF) [file pone.0278912.s004.tif]

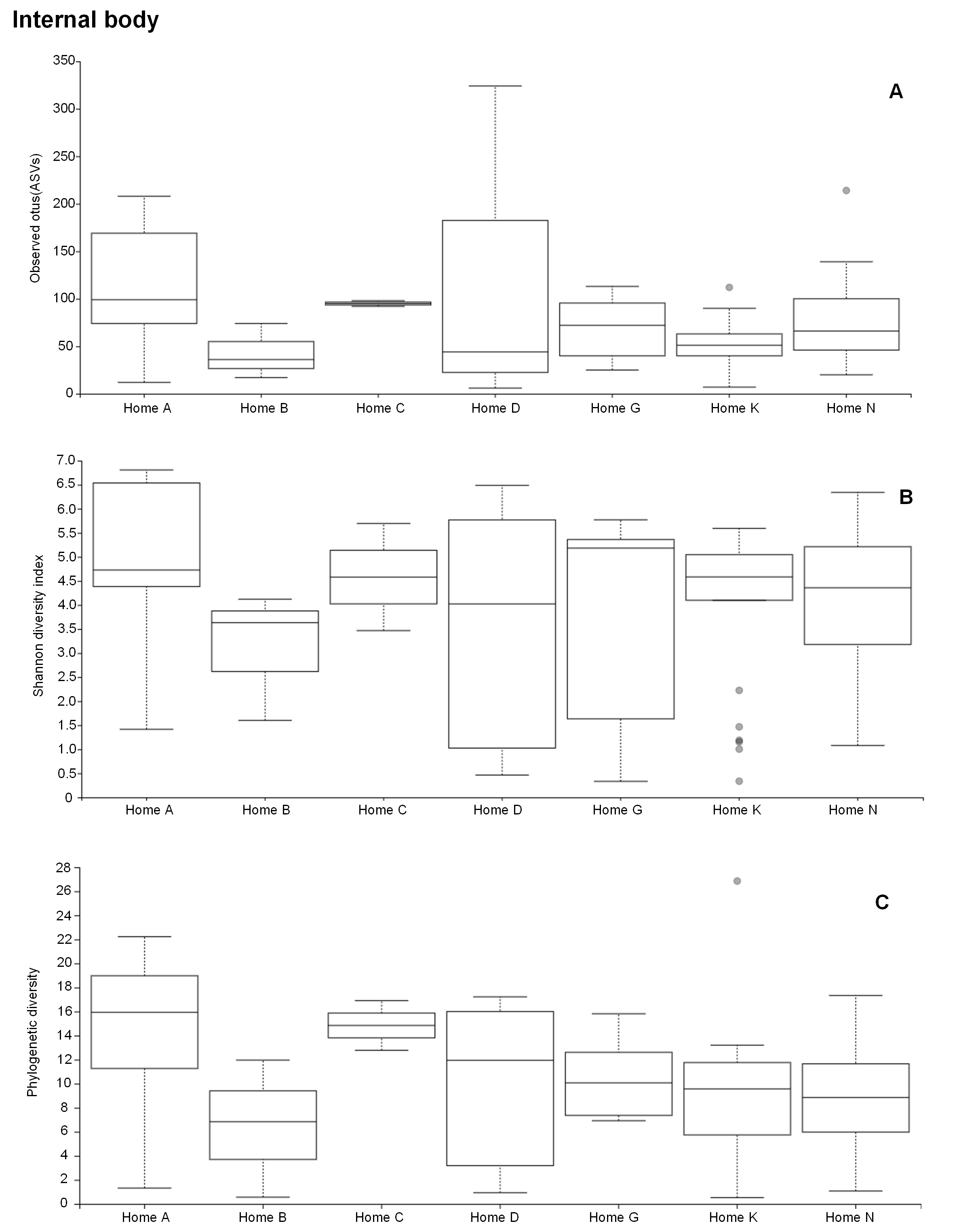

Supplement: S3 Fig — (A) Observed OTUs, (B) Shannon diversity and (C) Faith’s phylogenetic diversity. (TIF) [file pone.0278912.s005.tif]

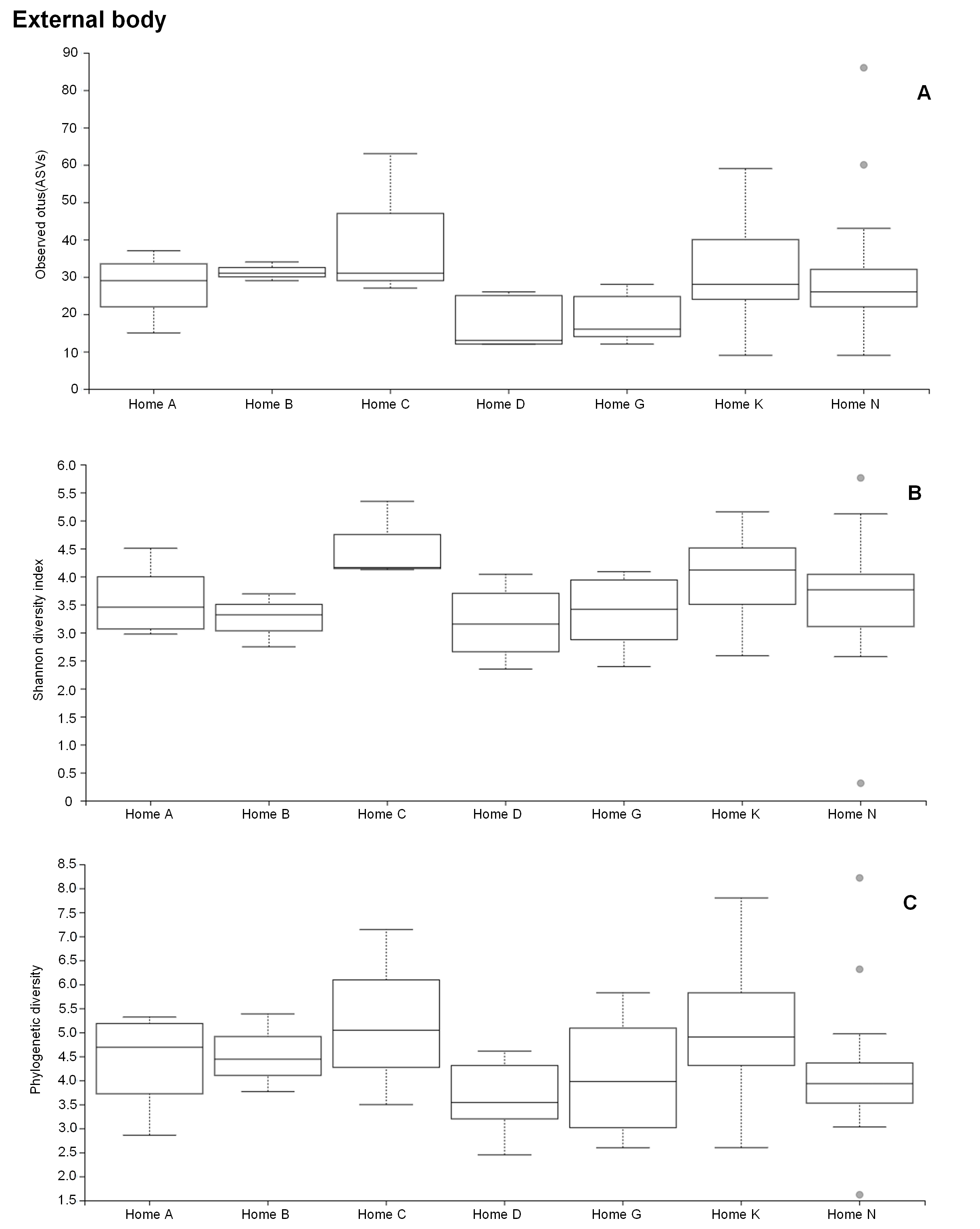

Supplement: S4 Fig — (A) Observed OTUs, (B) Shannon diversity and (C) Faith’s phylogenetic diversity. (TIF) [file pone.0278912.s006.tif]

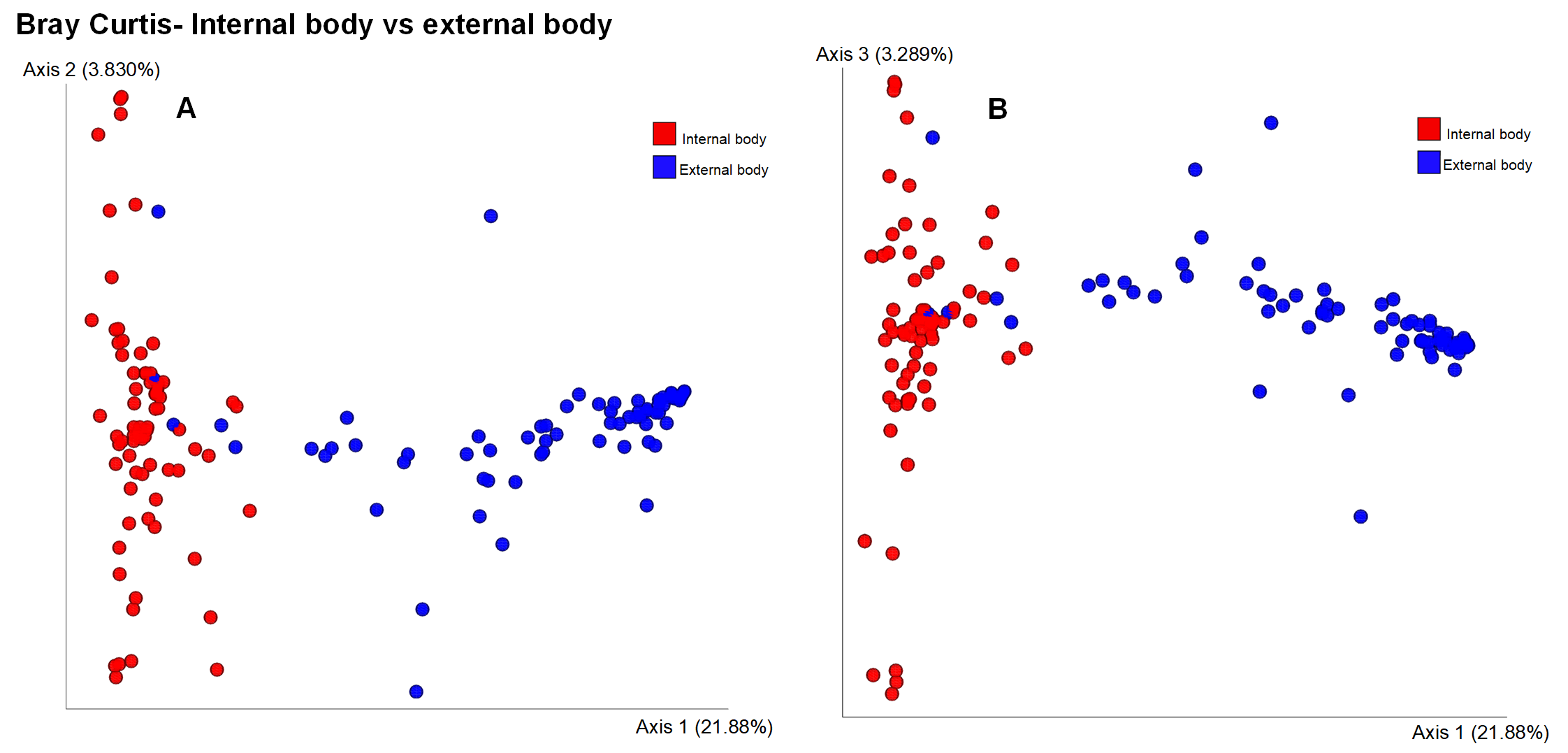

Supplement: S5 Fig — Analysis was based on the Bray Curtis metric. (TIF) [file pone.0278912.s007.tif]

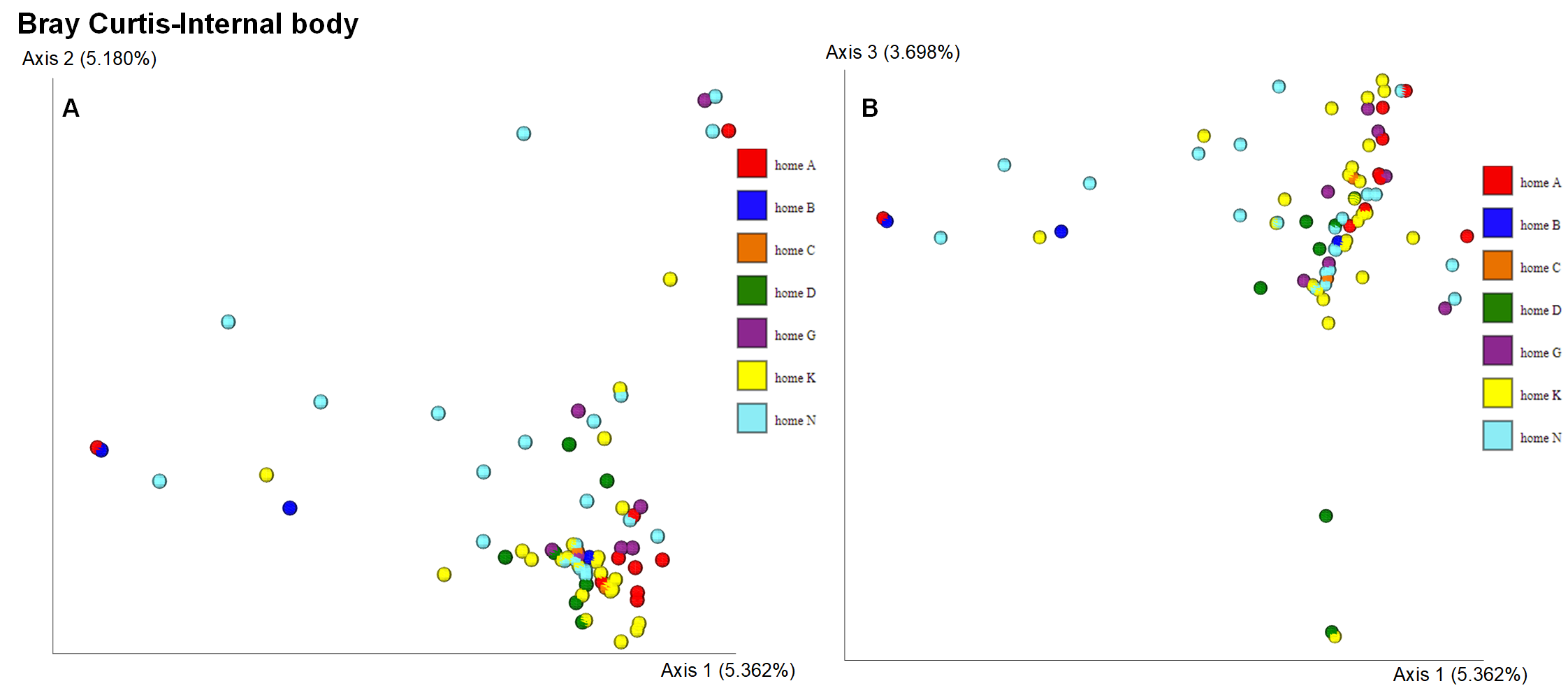

Supplement: S6 Fig — Analysis was based on the Bray Curtis metric. (TIF) [file pone.0278912.s008.tif]

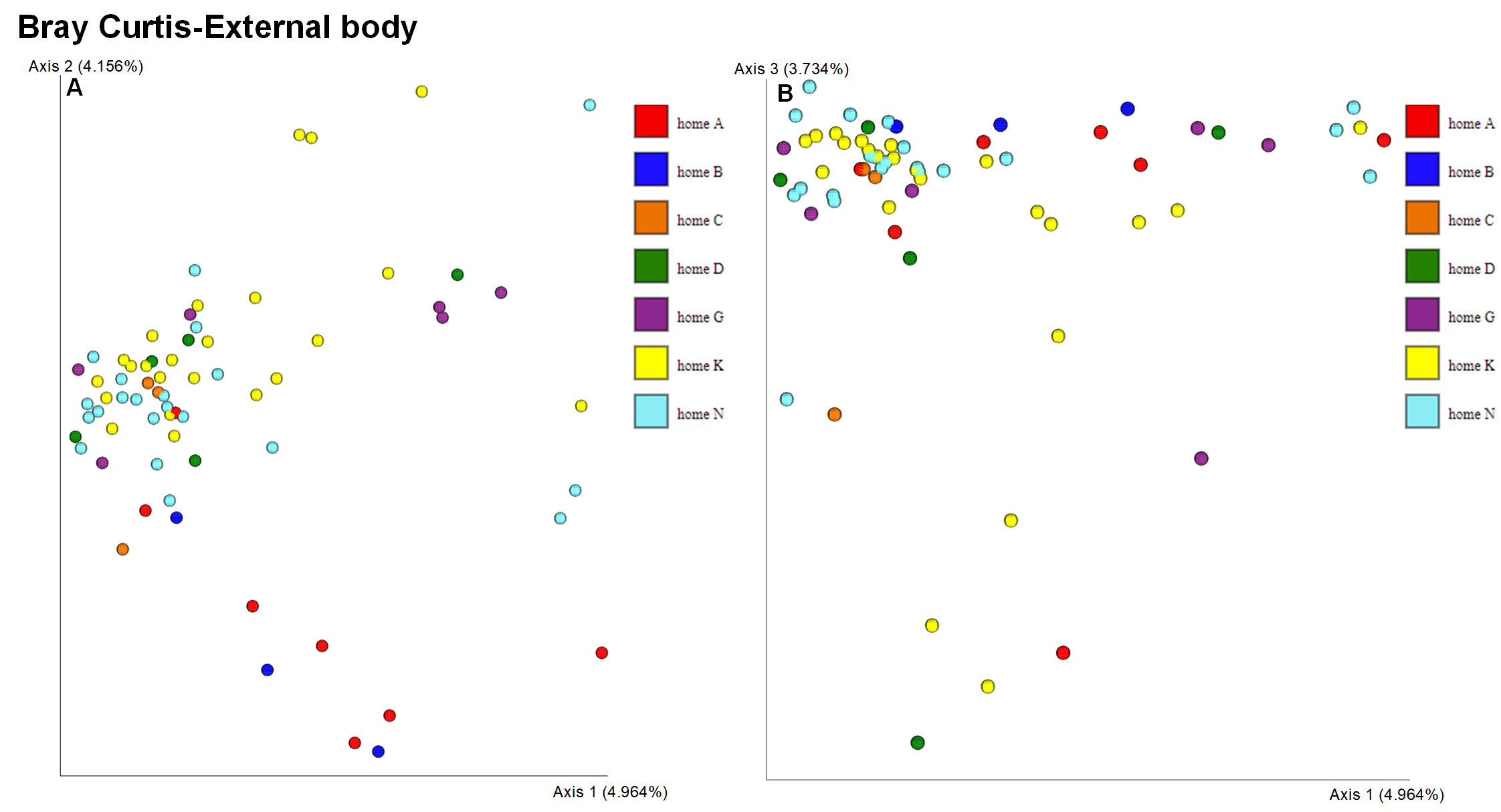

Supplement: S7 Fig — Analysis was based on the Bray Curtis metric. (TIF) [file pone.0278912.s009.tif]

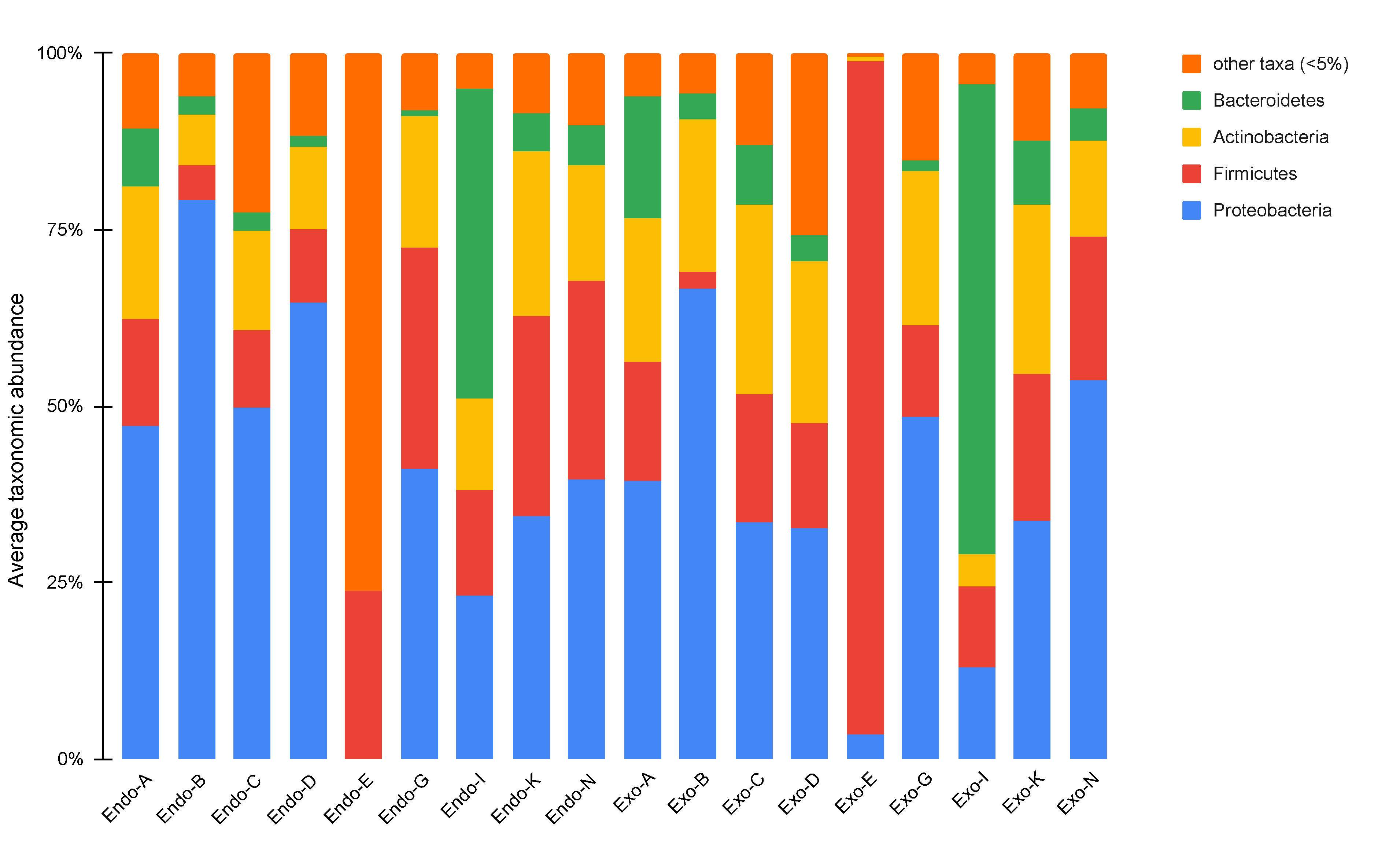

Supplement: S8 Fig — ‘Other’ group represents all taxa with relative abundance below 5%. (TIFF) [file pone.0278912.s010.tiff]

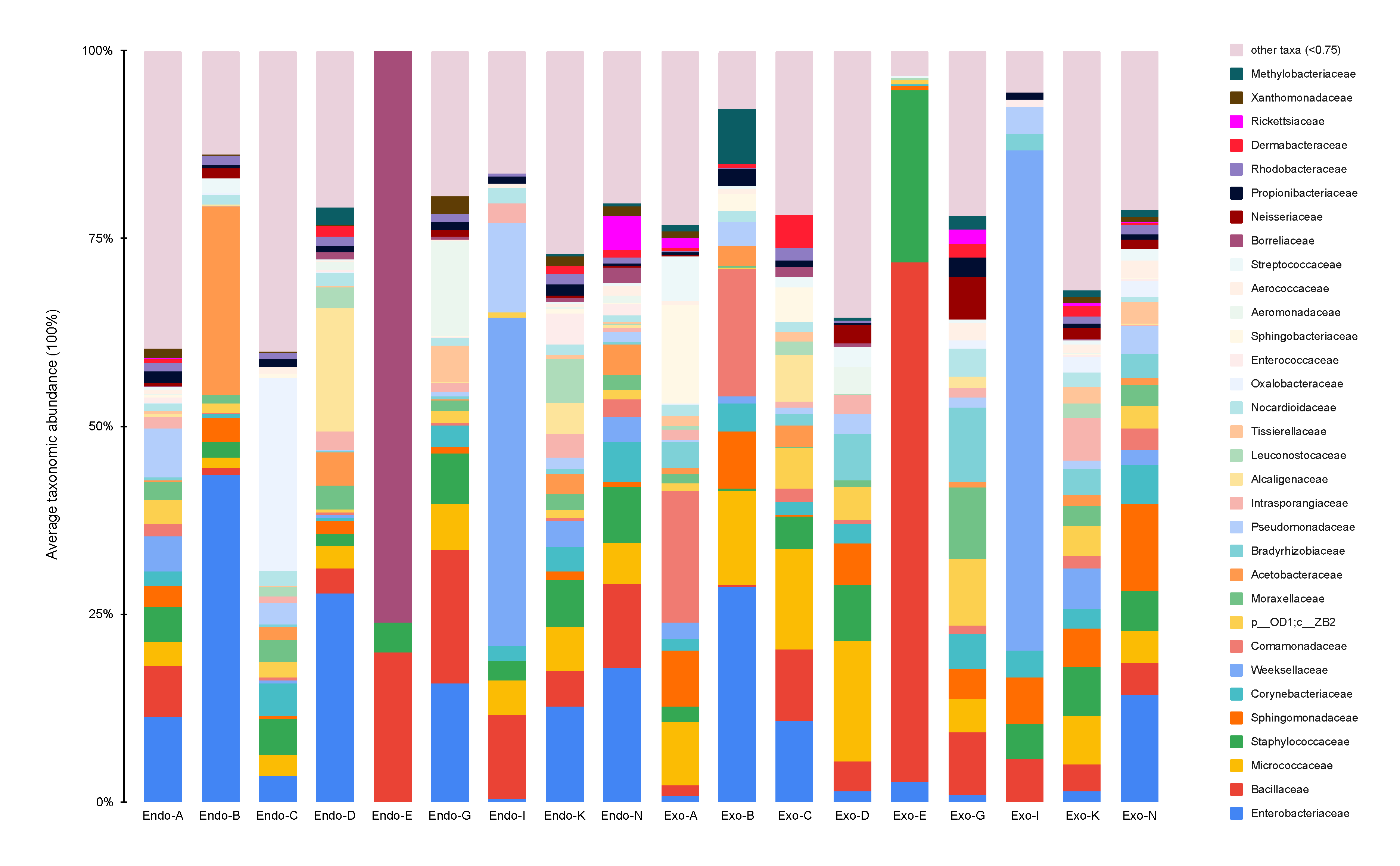

Supplement: S9 Fig — ‘Other’ group represents all taxa with relative abundance below 0.75%. (TIF) [file pone.0278912.s011.tif]

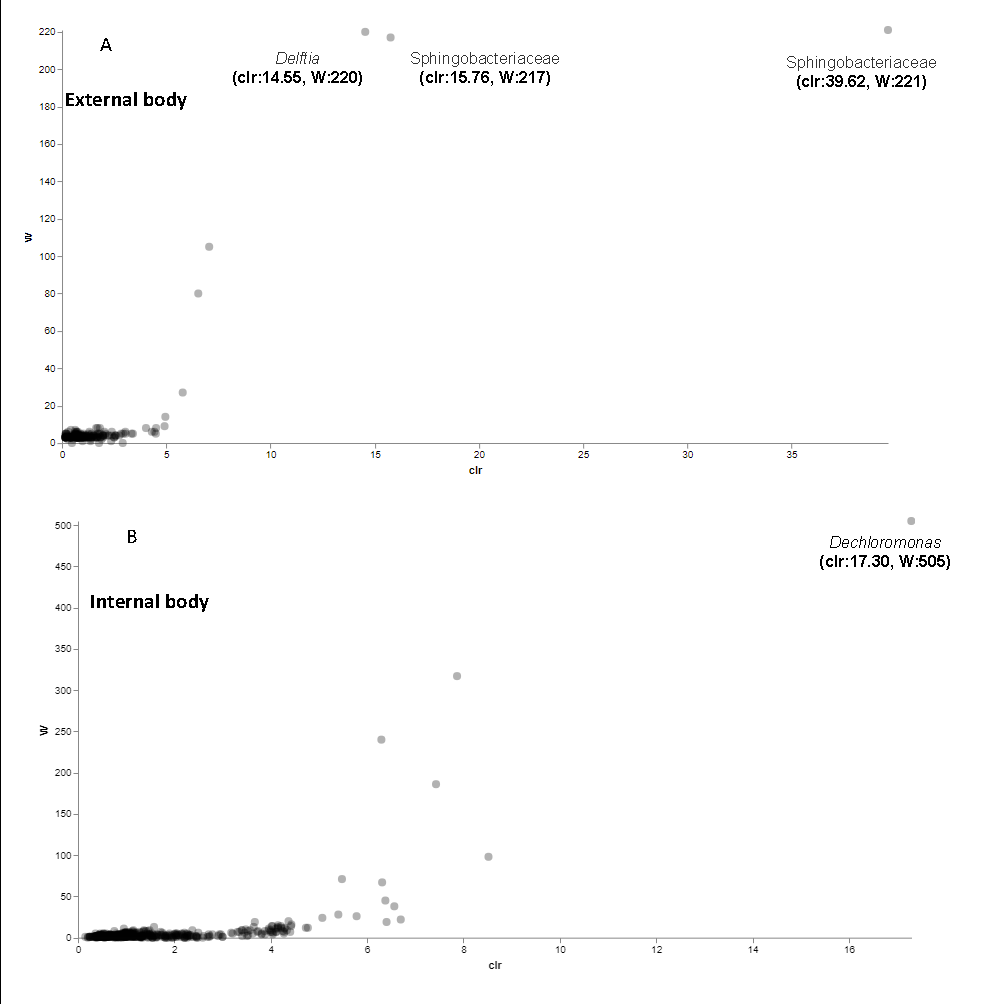

Supplement: S10 Fig — ANCOM differential abundance testing result for (A) external body and (B) internal body. Significantly differing taxa from ANCOM differential abundance testing resulted and the percentile abundance of taxa by group are shown in S2 Table. QIIME2 was used for ANCOM analysis. (TIF) [file pone.0278912.s012.tif]
